# Study protocol - the Australian National Child hearing Health Outcomes Registry (ANCHOR): collecting and connecting national data into a child deafness Learning Health System

**DOI:** 10.1186/s12913-026-14123-y

**Published:** 2026-03-04

**Authors:** Valerie Sung, Libby Smith, Kayla Elliott, Jing Wang, Rachael Beswick, Teresa Y. C. Ching, Hollie Feller, Samantha Harkus, Karolina Kluk, Kelvin Kong, Karen Liddle, Lauren McHugh, Catherine M. McMahon, Isabel O’Keeffe, Amie O’Shea, Jermy Pang, Bonny Parkinson, Ann Porter, Zeffie Poulakis, Emily Shepard, Daisy Shepherd, Patricia Van-Buynder, Philip Waters, Greg Leigh, Melissa Wake, and the ANCHOR Collaborative Group.

**Affiliations:** 1 Department of Paediatrics, University of Melbourne, Royal Children’s Hospital, 50 Flemington Road Parkville, Melbourne, VIC 3052 Australia; 2 Murdoch Children’s Institute, 50 Flemington Road, Parkville, VIC 3052 Australia; 3 The Royal Children’s Hospital, Centre for Child Community Health, Parkville, VIC 3052 Australia; 4 Queensland Government Department of Health, Brisbane, QLD 4001 Australia; 5 NextSense Institute, NextSense, Sydney, NSW 2109 Australia; 6 Macquarie School of Education, Macquarie University, Sydney, NSW 2000 Australia; 7 UsherKids Australia, Mordialloc, VIC 3195 Australia; 8 Hearing Australia, Sydney, NSW 2109 Australia; 9 Department of Linguistics, Faculty of Medicine Health and Human Sciences, Macquarie University, Sydney, NSW 2109 Australia; 10 Manchester Centre for Audiology and Deafness, The University of Manchester, Manchester, M15 6BH UK; 11 University of Newcastle, Newcastle, NSW 2300 Australia; 12 Macquarie University, Sydney, NSW 2109 Australia; 13 Children’s Health Queensland Hospital and Health Service, South Brisbane, QLD 4101 Australia; 14 The University of Queensland, St Lucia, Brisbane, QLD 4072 Australia; 15 HEAR Centre, Faculty of Medicine and Health Sciences, Macquarie University, Sydney, NSW 2109 Australia; 16 National Acoustic Laboratories, Sydney, NSW 2109 Australia; 17 Institute for Health Transformation, Deakin University, Geelong, VIC 3220 Australia; 18 School of Health & Social Development, Deakin University, Geelong, VIC 3220 Australia; 19 Macquarie University Centre for the Health Economy, Macquarie University, Sydney, NSW 2109 Australia; 20 Department of Economics, Macquarie Business School, Macquarie University, Sydney, NSW 2109 Australia; 21 Aussie Deaf Kids, Brunswick Heads, NSW 2483 Australia; 22 Department of Optometry & Vision Sciences, The University of Melbourne, Parkville, VIC 3052 Australia; 23 Deaf Victoria, Collingwood, VIC 3066 Australia

**Keywords:** Child hearing health, National registry, Learning Health System, Deaf and hard of hearing children, Community engagement, Data linkage, Equity

## Abstract

**Background:**

Despite early identification of deafness through universal newborn hearing screening, deaf and hard of hearing children can still face inequitable challenges in their development and life opportunities. Large scale registries, collecting standardised information on all individuals in a population with the relevant condition, can reveal variations in practices, processes, and outcomes, and identify targets for improvement. For childhood deafness, where care delivery spans multiple service sectors, data linkage is the only feasible way to bring data together. Data linkage also minimises the burden and costs of data entry by service providers, increasing future sustainability of large-scale research datasets.

**Methods:**

The Australian National Child Hearing Health Outcomes Registry’s (ANCHOR) mission is to collect and connect child hearing health information into a Learning Health System to drive research and service delivery improvements. Its vision is to give all deaf and hard of hearing children the best opportunities to reach their full potential and live healthy, fulfilled lives. ANCHOR aims to (1) Map Australia’s child hearing services and datasets, and determine the costs of establishing and maintaining ANCHOR nationwide; (2) Create a single cross-state data system, starting with two states, as a blueprint for national extension; and (3) Develop a Core Outcome Set to measure what matters to children, young people, families, and services. ANCHOR brings together expertise in child hearing health, education, lived experience, and research, with community engagement as its guiding principle.

**Discussion:**

ANCHOR will be a Learning Health System and national platform to facilitate population-based research for deaf and hard of hearing children. ANCHOR’s methodology, using community engagement at its heart, will also serve as a prototype for other health conditions using data linkages to sustainably capture large-scale data, to ensure data typically missing from vulnerable families can be included. Ultimately, ANCHOR will generate robust, actionable evidence to optimise service delivery models and equity of access for deaf and hard of hearing children and their families. This paper provides an overview of the established and evolving methodologies of the ANCHOR project to achieve its aims.

**Supplementary Information:**

The online version contains supplementary material available at 10.1186/s12913-026-14123-y.

## Introduction

Childhood deafness can have profound impacts on communication, development, quality of life, adult opportunities, and service needs. Over the last 25 years, seismic advances have been made in reducing the age at identification of childhood deafness and in the development of new interventions and support pathways for deaf and hard of hearing (DHH) children and families [1, 2]. Newborn children in Australia are routinely screened for hearing loss through universal newborn hearing screening (UNHS) programs in every state and territory [3, 4]. Australia invests in UNHS at a yearly cost of at least AUD$22 million [5]. In principle, every DHH child has free and early access to hearing screening, diagnosis, and support, including hearing devices, cochlear implantation, and early intervention programs, to help them achieve optimal communication and developmental outcomes [1, 6, 7]. Australia enviably exceeds international targets for the proportion of infants screened by 1 month (> 97%) [8].

However, this achievement represents only the first step in delivering a successful hearing health program. As Muir Gray quotes, “all screening programs do harm; some do good as well” [9]. The success of any screening program should be measured by improved outcomes that result directly from effective support and management. Despite the remarkable advances in the early detection of hearing loss, research suggests DHH children continue to face greater developmental challenges than their hearing peers [10–12]. The Longitudinal Outcomes of Children with Hearing Impairment (LOCHI) study showed that language scores for the participant children, including those with additional health needs, were on average more than one standard deviation lower than expected for typically hearing children [2]. Analysis demonstrated that, although UNHS maximises the opportunity for early intervention, not all children who were screened commenced amplification early and not all unscreened children commenced amplification late [13]. Factors such as cognitive ability, parental level of education, socioeconomic status, age at amplification, or cochlear implantation may explain part but not all of the variance in developmental outcomes. This raises an important question: could the persistent disparities between children who are DHH and those who are not be due to inequitable access to supports, interventions, and services – inequities that could be addressed if properly identified? Can we identify the gaps in services for children who need them the most – for example, Aboriginal and Torres Strait Islander children, who experience disproportionately high rates of otitis media-related deafness in childhood [14]? Are we providing the right services for DHH children, including those from the deaf community, who may have different support needs? In this paper, the lower case ‘d’ (‘deaf’) is used as an all-encompassing term to acknowledge different identities with which a deaf or hard of hearing person may identify and the different means by which they may communicate, including Auslan.

Australia as a whole does not have complete, accurate data on whether diagnostic audiology or hearing amplification targets are achieved for all children because not all states consistently collect the data, and there is no national system to identify children who may not access services. There is currently no systematic collection of data on the developmental, educational, or well-being outcomes of children detected by UNHS. Importantly, outcomes related to the language skills of children who communicate by Australian Sign Language (Auslan) may not be identified or measured. We need to collect the right data on outcomes of UNHS at scale – information that is critical for understanding why DDH children may experience inequitable outcomes, understand true bias in healthcare access, and for evaluating the impact and cost-effectiveness of UNHS and service delivery [15].

Nelson et al. noted: “Large scale collection and analysis of data on patients’ experiences and outcomes have become staples of successful health systems worldwide” [16]. Whether termed registries, clinical databases, or quality improvement programs, their hallmark is standardised information including all individuals in a population with the relevant condition, to reveal variations in practices, processes, and outcomes, and identify targets for improvement. But maintaining registries can be expensive and time-consuming. Cost and burden can be minimised, and completeness and accuracy enhanced, via data linkage mechanisms uniting data from disparate sources [17, 18]. Moreover, for childhood deafness, where practices, processes, and outcomes all span multiple service sectors, data linkage is the only possible way to bring data together. In Australia for example, the Generation Victoria study, the nation’s largest investment in population child health costing more than AUD$50 million, is linking a whole-state cohort derived from the UNHS sampling frame (99% reach) with federal and state services, health, and administrative datasets, including newborn, maternal and child health, and hospital data [19]. In the state of Queensland, health and education departments are linking large population datasets to understand children’s outcomes, including hearing [20]. These prime works-in-progress are navigating the technical, legal, and consent issues surrounding data linkage.

Data linkage cannot occur successfully if services do not collect the same outcomes data and interpret the data in the same way. Currently, outcomes for DHH children in Australia are collected and measured heterogeneously. There is a need to enable programs, services, and research studies to collect a core set of shared outcomes data for Australian DHH children and young people. Ideally, such outcomes data should be relevant, accurate, and meaningful to DHH children and young people, their families, service providers, clinicians, and stakeholders. The Core Outcome Set (COS) offers a potential solution to the challenges created by inconsistent outcomes and measurement tools. A COS is an agreed minimum set of outcomes that should be measured and reported for a specific condition [21]. It does not limit the outcomes that should be measured or recorded, rather it mandates that the COS is included as a minimum standard [21]. The COS should be developed in partnership with the deaf community and with Aboriginal and Torres Strait Islander peoples to ensure cultural appropriateness, understanding and meaningful application.

With the ubiquitous use of electronic health data and the increasing maturity of data linkage mechanisms in Australia, the opportunity exists to develop a national mechanism to systematically track outcomes of all Australian DHH children, to create a Learning Health System that enables research and quality improvement. Many well-honed, high-quality, individual mature datasets are now in place for DHH children in Australia (see Additional File 1). No single dataset can tell the full story. By flexibly combining, linking, or federating datasets - using shared identifiers, data models, terminology, and core outcomes - a connected national system can deliver comprehensive insights while avoiding duplication and inefficiencies. The Australian National Childhood Hearing Health Outcomes Registry (ANCHOR), funded by Australia’s National Health and Medical Research Council (NHMRC), is being established to achieve this goal. ANCHOR’s vision is to give all deaf and hard of hearing children the best opportunities to reach their full potential and live healthy, fulfilled lives. ANCHOR’s mission is to collect and connect child hearing health information into a national data system and research platform to drive better care.

This protocol paper describes the novel methodology we are using to establish ANCHOR as a national Learning Health System for DHH children age 0–18 years in Australia. ANCHOR has three aims with three methodological streams:

1. Map and understand Australia’s child hearing services and datasets, and determine the potential costs of establishing and maintaining ANCHOR nation-wide;
2. Create a single cross-state data system through data linkage: starting with two states (Victoria and Queensland) as a blueprint for national extension; and.
3. Develop a Core Outcome Set to measure what matters to children, young people, families, and services, against which to measure continually improving practice and service delivery over time.

## Methods

### Community and public engagement as the guiding principle of ANCHOR

**Fig. 1:**
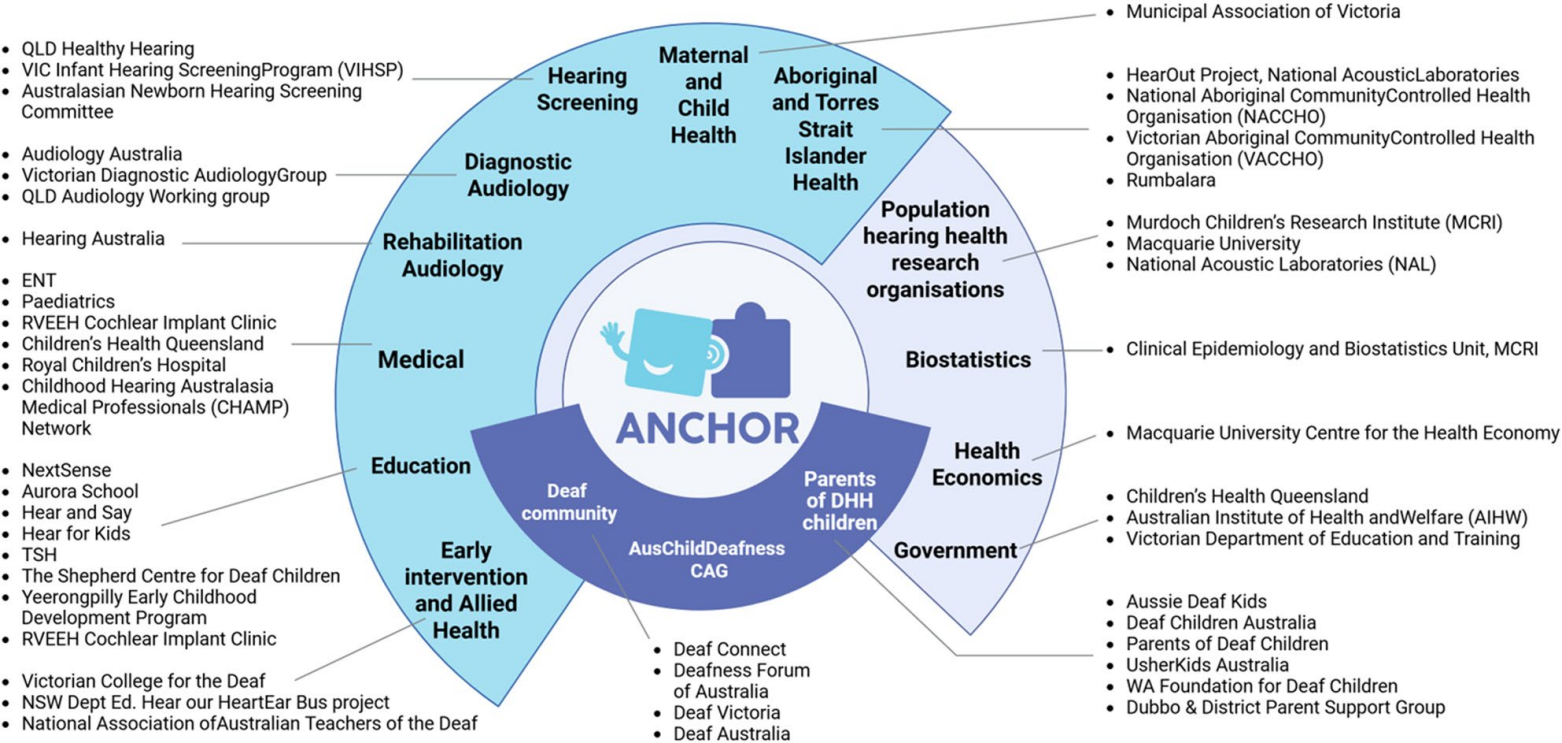
Stakeholders of ANCHOR (see Additional File 2 for Glossary of organisations)

The ANCHOR methodology is founded on a strong commitment to community and public engagement. The ANCHOR Investigator Team, consisting of 20 leading experts in child hearing health, are co-designing ANCHOR from its inception with the ANCHOR Advisory Committee and the Australian Childhood Deafness Research Community Advisory Group (AusChildDeafness-CAG), established through ANCHOR’s funding (Fig. 1). The ANCHOR Advisory Committee includes a diverse group of > 100 child hearing health stakeholders from > 15 professional groups including audiologists, teachers of the deaf, doctors, researchers, deaf advocates, parent representatives, early intervention providers, educational professionals, Aboriginal and Torres Strait Islander and government representatives. The AusChildDeafness-CAG is chaired and led by families with living/lived experience. Recruitment for the AusChildDeafness-CAG membership was led by a core group of four parents who independently selected 15 members with lived experience from diverse backgrounds, including both hearing and DHH parents of different ages, whose DHH children are of different ages, hearing profiles and cultural backgrounds, and who communicate through oral and/or sign languages. The membership also includes young people aged 16–26 years who are DHH. Members of the AusChildDeafness-CAG are supported to undergo training aiming to increase their understanding of health and medical research and the value and goals of consumer and community involvement. Following the Victorian Comprehensive Cancer Centre Model of Consumer Engagement [22], AusChildDeafness-CAG members can contribute to the ANCHOR program by informing, consulting, involving, and partnering with the project team or by CAG-led activities. All AusChildDeafness-CAG members receive financial remuneration for time spent involved in ANCHOR program activities.

We have built and are continuing to develop relationships with stakeholders to ensure community and public voices are central to the co-designed development of ANCHOR. This approach ensures meaningful community engagement, respects consent, privacy, cultural, ethical and security considerations; facilitates effective communication of research ideas and processes; and helps translate findings into action. We are accounting for the necessary financial resources to meaningfully engage consumers and their communities, and to provide adequate administrative support. We will ensure analysed data are relevant, clearly interpreted in terms all stakeholders can understand, and are shared through appropriate community channels. We will seek feedback on the appropriateness and accuracy of reports before broad dissemination, and acknowledge all representative stakeholder co-authors in any resulting publications. These steps will increase the likelihood of more effective practice implementation going forward. We are ensuring continuous learning by reflecting on the effectiveness of our processes, and on the roles and performance of both researchers and community members. We are using culturally appropriate methods to engage both DHH Auslan users and Aboriginal and Torres Strait Islander communities as we expand our stakeholder connections. Specifically, we are partnering with representatives from the deaf community and from Aboriginal and Torres Strait Islander communities, and with researchers with deep links to these communities, in protocol development. Given the negative historical experiences of research and data collection involving the deaf community and Aboriginal and Torres Strait Islander populations, we aim to prioritise innovation through genuine collaboration and meaningful engagement. This approach seeks to enhance community responsiveness and ultimately improve outcomes for families.

We are conducting quarterly investigator meetings and biannual Advisory Committee meetings, with specified reporting timeframes six monthly. We have formed working groups to collaboratively develop the methodology for each of ANCHOR’s aims, and regularly engage with the AusChildDeafness-CAG through online meetings and email correspondences for inputs in all aspects of ANCHOR. We are ensuring research partnerships are conducted fairly, efficiently, and effectively.

### Aim 1: Map and understand Australia’s child hearing services and datasets, and determine the potential costs of Establishing and maintaining ANCHOR nation-wide

To inform the development of a prototype ANCHOR, we are conducting a national mapping survey to understand the current landscape of child hearing health services and related datasets across Australia. The mapping survey also aims to determine the magnitude of costs required to establish and maintain ANCHOR at a national level. This stream of work aims to assess the future feasibility of integrating data into a single, connected national system.

#### Survey development

The online survey is co-designed by a Working Group comprising 37 stakeholders from diverse professional and service backgrounds. The questions for the cost-analysis were developed in consultation with Macquarie University’s Centre for Health Economy. The survey defines “hearing health services” as those providing care to children aged 0–18 years who are DHH. These include services related to hearing screening, diagnosis, rehabilitation, medical care, Aboriginal and Torres Strait Islander health, maternal and child health, early intervention (oral and sign), education, parent support, and deaf advocacy. “Databases” refer to systems used for clinical, administrative, or research purposes for DHH children.

Survey questions collect the following information (see Additional File 3 for full survey content):

- Service name and type.
- Funding sources.
- Referral pathways.
- Data and outcome measures (including child age, data collection methods, timing of entry, and format).
- Equipment and software used for data storage.
- Details of any existing databases.
- Data linkage mechanisms in place.
- Consent processes for data use.
- Legal compliance for data sharing.
- Costs: current staffing and time involved in data collection and entry, wage and salary expenses, costs of data storage equipment and software used.
- Identification of other similar services or databases.
- Contact details and consent for future follow-up.

A follow-up online stakeholder survey will be conducted following the development of the COS (Aim 3) to assess the anticipated changes in data collection processes on personnel, equipment, and software costs.

#### Survey distribution and data collection

Survey distribution is being conducted via multiple channels, including ANCHOR stakeholder networks, the Australasian Newborn Hearing Screening Committee and its contacts, professional hearing health conferences, and snowball sampling methods. Invitations to participate are sent by email, containing a link to the online survey hosted on REDCap. Each organisation is asked to nominate one representative to complete the survey, with an estimated completion time of 1–2 h.

To maximise response rates, the ANCHOR research team follows up on incomplete surveys by contacting the nominated individual listed in the REDCap submission. Respondents are also offered the option to complete the survey during a guided online meeting with a member of the research team, allowing for real-time clarification and support.

#### Data analysis

We anticipate reporting on approximately 100 databases across Australia. Descriptive statistics will be used to summarise the results. Continuous variables will be presented as means and standard deviations, while categorical variables summarised as frequencies and proportions.

For the cost analysis, we will estimate the anticipated costs of developing, implementing, and maintaining ANCHOR. The costs will be divided by the number of individuals receiving hearing and support services annually, and the volume of hearing and support services provided, to estimate the data cost per DHH child. Additionally, we will supplement these cost estimates with costs associated with data linkage, storage, and the Data Governance Committee (from Aim 2). We will project future costs based on the data system growth rate, theoretical capacity, and equipment and software upgrades, drawing on expert opinion.

There are three approaches to valuing data: a market-based approach, a dimensional approach, and an economic approach [23]. A market-based approach assumes that the value of data is equal to the revenue it may generate. However, this approach would likely undervalue ANCHOR, as access to data will be provided either for free or on a cost-recovery basis. A dimensional approach involves describing the data in terms of attributes (e.g. ownership, cost, utility, age, privacy, data quality, and volume and variety) and using stated preference survey methods to assign value each attribute. However, this approach generally requires a large sample size (e.g. 400 hearing researchers who may use the data) and tends to focus on the value perceived by survey respondents, rather than the broader benefits to DHH children, their families and society [24]. An economic approach, on the other hand, involves using models to assess the economic impact, including financial, health, social and policy impacts [23]. A health economic approach is commonly used by policymakers to inform health policy or funding decisions, making it particularly suitable to valuing ANCHOR. We will develop economic models of policy case studies regarding the provision of health services to DHH children. These case studies will be selected through a combination of literature review and expert opinion, focusing on those with significant uncertainty regarding cost-effectiveness, where ANCHOR could reduce this uncertainty and influence policy or funding decisions. Examples of potential case studies include screening programs for pre- and school-age children, providing hearing aids for children with mild or unilateral hearing loss, and increasing the frequency of hearing services [4, 25]. The economic models will estimate the impact of these interventions on the quality of life of DHH children, health service use and associated costs. The value of ANCHOR will be estimated as the expected reduction in the probability of making a wrong policy or funding decision multiplied by the average consequence of being ‘wrong’. Specifically, we will estimate the Expected Value of Sample Information (EVSI) using non-parametric, regression-based methods [26], assuming a willingness to pay threshold of $60,000 per Quality Adjusted Life Year gained [27]. If the aggregate EVSI across all case studies exceeds the anticipated cost of ANCHOR, this indicates there will be a net benefit in developing ANCHOR. It is important to note that this estimate will represent a lower-bound value of ANCHOR, as not all potential studies using ANCHOR will be valued, and ANCHOR could potentially inform policy and funding decisions globally.

### Aim 2: Create a single cross-state data system through data linkage: starting with two states (Victoria and Queensland) as a blueprint for national extension

To develop a prototype ANCHOR, we are conducting a proof-of-concept data linkage project in two Australian states - Victoria and Queensland. This work involves linking existing hearing health datasets and outcomes datasets across the continuum of care, including newborn hearing screening, diagnosis, rehabilitation, early intervention, education, and parent support services.

Victoria and Queensland were selected for this initial phase due to the existence of two large statewide datasets: (1) The Victorian Childhood Hearing Longitudinal Databank (VicCHILD), which collects detailed outcomes data on children identified through UNHS in Victoria [28], and (2) The Queensland QChild database, which tracks program-level outcomes from UNHS through to early intervention.

#### Data governance and FAIR principals

ANCHOR is being developed in alignment with FAIR principles, ensuring that all data are Findable, Accessible, Interoperable, and Re-usable [29]. A Data Governance Committee has been established to oversee the ethical and secure application of these principles.

To facilitate secure and ethical data sharing, we are working in close collaboration with a stakeholder Working Group of five key child hearing health partners in Victoria and Queensland. The group meets jointly and individually to address critical issues including data governance, consent pathways, and bi-directional data flow between services and databases.

#### Consent and data sharing mechanisms

**Fig. 2:**
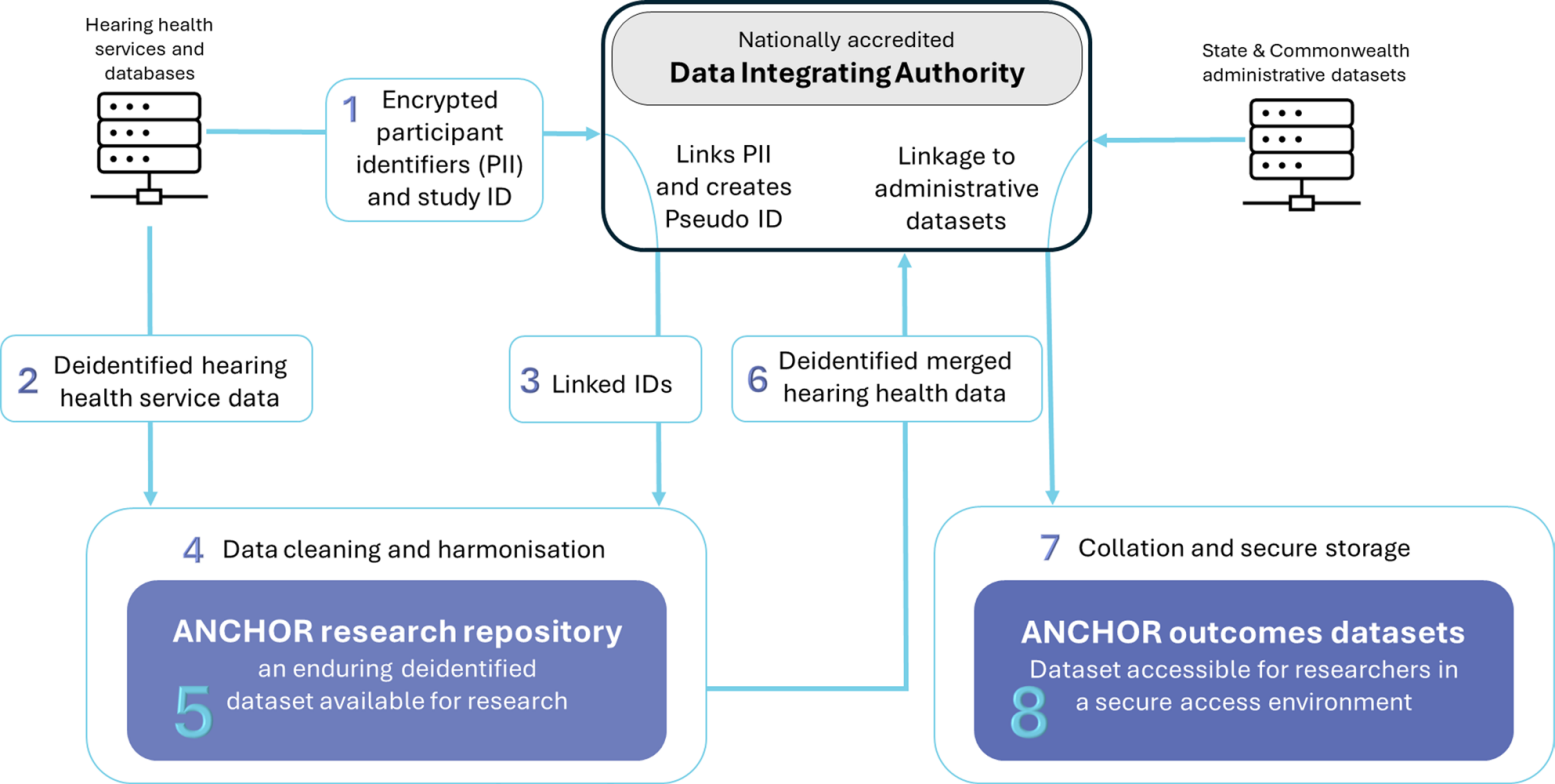
Conceptual diagram of ANCHOR phase one – anonymised data repository

We are co-developing brief, standardised intake consent statements that can be adopted by major hearing health services (Fig. 2) to enable prospective data sharing and linkage. These statements ensure transparency for families about how their personal information may be used and protected. All data will be stored under strict privacy controls in secure environments, such as the SURE (Secure Unified Research Environment) platform, hosted for the Australian Institute of Health and Welfare (AIHW), and the VALT (Victorian data Access Linkage Trust) platform, hosted by the Centre for Victorian Data Linkage (CVDL).

#### Date linkage model: phase one

Given that some datasets in Victoria and Queensland currently lack explicit research consent, the data linkage is being implemented in two phases. Phase One, supported by current ANCHOR funding, focuses on linking de-identified data under strict privacy protocols applying the separation principal to ensure personal identifiers remain completely separate from content data. To support secure and accurate data linkage, we have established a multi-step protocol involving accredited Data Integrating Authorities, participating data custodians, and relevant state and commonwealth datasets.

The data linkage workflow is illustrated in Fig. 2 and described below:

1. Transfer of Personal Identifying Information (PII) to the Integrating Authority: Each participating data custodian will assign a unique study-identification number (ID) to all eligible children in their database. The PII and corresponding study-ID will be transferred to an accredited Data Integrating Authority via secure channels.
2. Transfer of de-identified data to the ANCHOR Repository: In parallel, each service will transmit de-identified hearing health data, tagged with the same study-ID, directly to the ANCHOR research repository. This separation of PII from health data will maintain confidentiality while enabling subsequent linkage.
3. Generation and transmission of the pseudo-ID: The Integrating Authority will generate a pseudo-ID for each child, mapped to the associated study-ID provided by each service, and securely transmit these linked IDs to the ANCHOR repository.
4. Data Cleaning and Harmonisation: The ANCHOR repository will apply systematic data cleaning and convert all incoming datasets into a common model, using standardised vocabularies and coding systems to ensure interoperability.
5. Data Merging and Resource Formation: Using the pseudo-ID, the ANCHOR repository will merge datasets into a single anonymised research resource containing variables such as exposure (e.g. hearing loss severity), service variables (e.g. hearing aid fitting, type and timing of intervention) and outcomes (e.g. developmental and educational).
6. Linkage to administrative datasets: The Integrating Authority will link the ANCHOR data to additional State and Commonwealth datasets, including education records and child development data, to enable longitudinal outcome analyses.
7. Collation and secure storage: Linked datasets will be stored within a secure online access environment managed by the Integrating Authority.
8. Researcher access: Approved researchers will access the ANCHOR datasets within the secure environment to address defined research questions under strict governance and ethical oversight. Supporting materials, including a comprehensive data dictionary and a formal data access process will be provided.

The proposed Phase One ANCHOR dataset will not hold identifiable information. This limits its use for some types of future research.

#### Toward ANCHOR phase two: prospective consent and identifiable data linkage

Phase Two of ANCHOR will aim to implement prospective consent processes within hearing health services. In this phase, identifying information could be used, under appropriate ethical approvals, for richer, longitudinal data linkage. This would enable broader, ethically governed research access to more comprehensive datasets. To support this transition, ANCHOR will seek future funding to enable service-level changes that facilitate routine collection of research consent, supporting the implementation of the Phase Two model across additional jurisdictions.

#### Generalisability and national scalability

All processes developed in Victoria and Queensland are being designed with national scalability in mind. ANCHOR is working with data custodians and establishing partnerships with accredited Data Integrating Authorities at both the national (e.g. AIHW) and state levels (e.g. CVDL, Data Linkage Queensland). These partnerships will ensure that governance frameworks, technical infrastructure, and privacy protections are transferable and can support future expansion of ANCHOR across Australia.

### Aim 3: Develop a core outcome set to measure what matters to children, young people, families, and services

**Fig. 3:**
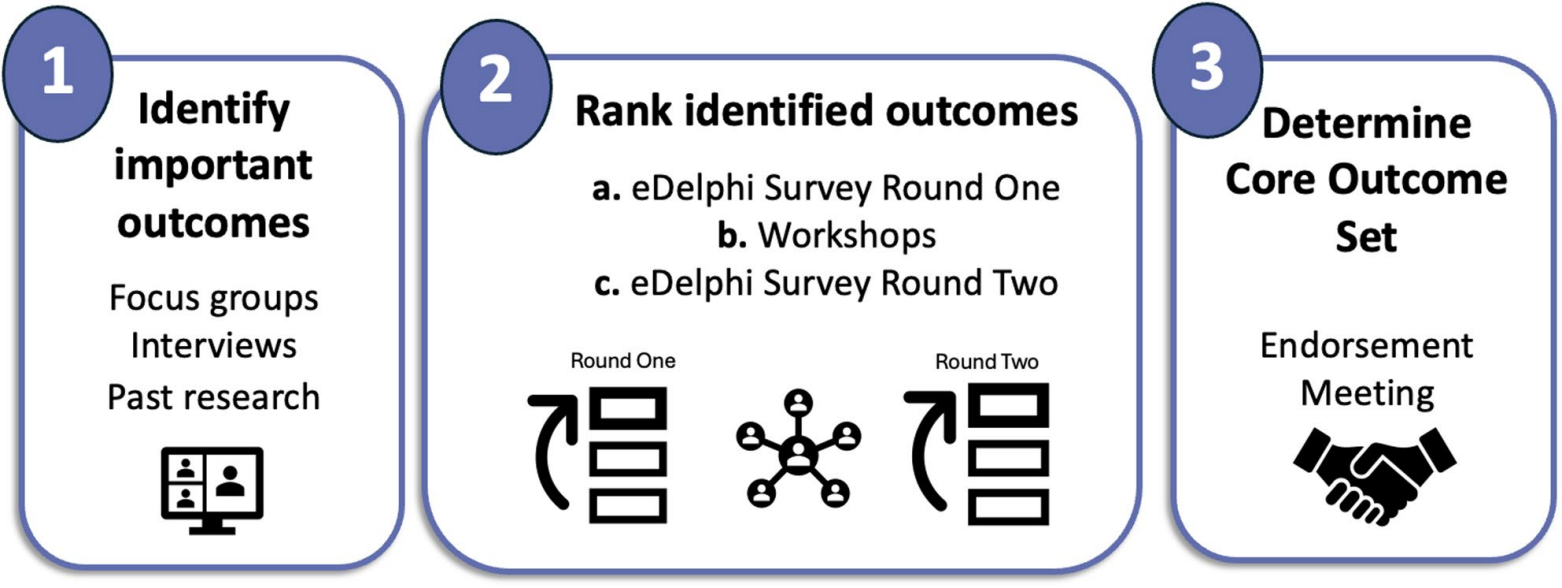
The three main steps for achieving a child deafness COS

To support successful future data linkage, we are developing a child deafness Core Outcome Set (COS) to encourage services and research studies to collect a standardised set of outcomes data. This minimum dataset will promote consistency across contexts and facilitate robust comparisons. To maximise feasibility and long-term sustainability, the COS must be relevant and meaningful to DHH children and young people, their families, service providers, clinicians, and other stakeholders. A dedicated Aim 3 Working Group has been established, consisting of 18 child deafness stakeholders (clinicians, researchers, educators, child deafness advocacy groups) and members of the AusChildDeafness-CAG. This group is co-designing the Aim 3 methodology with a particular focus on ensuring that the design of questions for interviews and focus groups, and methodologies to reach consensus on a COS, are culturally appropriate, sensitive, and effective. Figure 3 illustrates the three main steps of reaching a COS: (1) Generation of a ‘long list’ of outcomes from focus groups, interviews and existing evidence from the literature (systematic and scoping reviews); (2) Sorting outcomes into domains and ranking them through e-Delphi surveys and workshops; and (3) Endorsement meeting. This is an active process, and the methodology described below reflects the current approach at the time of this protocol publication. Further refinements are anticipated to ensure responsiveness to ongoing stakeholder feedback.

#### Step 1: Generation of a ‘long list’ of outcomes

To create a ‘long list’ of outcomes, we are capturing published outcomes from existing literature as well as family and stakeholder perspectives. In the United Kingdom (UK), the Prioritising Outcomes iN Childhood Hearing lOss (PONCHO) study, led by Manchester University, aims to determine the most important child hearing loss outcomes using the Core Outcome Measures in Effectiveness Trials (COMET) Initiative [30]. The PONCHO study has conducted a systematic review of existing child hearing outcomes collected in previous research studies [31]. To complement this work, we are conducting a rapid literature review of family and DHH people’s perspectives of important outcomes, along with interviews and focus groups with a range of families with different characteristics.

#### Rapid literature review methodology

Our review addresses the question: “What social, emotional, educational and mental health outcomes matter to Australian children and young people who are DHH and their families?”. The review focuses on qualitative or mixed-methods research conducted in Australia in the last 10 years (2014–2024) that reports on lived experience of deafness and includes parent/carer or young person perspectives. The reported outcomes from this rapid literature review, along with those identified from the focus groups and interviews, inform the e-Delphi Round One survey.

#### Focus groups / interviews methodology

We are inviting families and young people through ANCHOR stakeholders and social media to express their interest in participating through a REDCap online platform link [32, 33]. The link allows parents/caregivers or young DHH people to access the relevant information in English and Auslan, and register their interest to participate in various parts of the project, indicate preferences for focus group meeting times, and basic demographic information, child hearing loss details, interpreter needs. Purposive sampling ensures diversity across: (1) Parents/carers of DHH children aged up to 26 years old who are DHH, of different ages, hearing loss types and severities, from non-English speaking backgrounds (interpreters offered if needed) and of Aboriginal and Torres Strait Islander backgrounds; and (2) Young people aged 16 to 26 years old who are DHH, of different ages, hearing loss types and severities. The upper age limit of 26 –years reflects eligibility for free hearing healthcare in Australia, including free access to hearing aids and associated devices.

The Aim 3 Working Group is contributing to the design of questions for semi-structured interviews and focus groups for different participant groups and piloting them to ensure questions are sensitive and appropriate to address the research question of ‘what outcomes matter most to you and your family’. Focus groups and interviews are held online, or in person where feasible. Online focus groups are co-facilitated by a deaf researcher, audiologist, and parent of a DHH child, and involve not more than four families per focus group to ensure all focus group participants have adequate opportunity to express their views.

To ensure that we identify which outcomes matter most to the deaf community, we are consulting with representatives of deaf organisations, facilitated by a researcher with expertise in co-designed approaches. Author AO is a hearing researcher fluent in Auslan and with deep links to the Australian deaf community. The focus groups and the core outcome workshop are being led by community organisations sub-contracted to provide this in appropriate culturally safe and inclusive environments, and are conducted purely in Auslan. To enable nationwide participation, engagement is taking place online via video conferencing (e.g. Zoom) and workshopping tools (e.g. Miro).

To ensure the inclusion of parents and caregivers of DHH Aboriginal and Torres Strait Islander children, our recruitment methodologies are tailored to each community’s preferred and acceptable approaches. This acknowledges the requirement for proposals involving Aboriginal and Torres Strait Islander communities to be developed with communities, organisations, or reference groups, as appropriate. The research team is working with the local Aboriginal Community Controlled Health Organisation or reference committee in different states to ensure recruitment strategies are appropriate, and is applying for ethics approval modifications where required. We are consulting with the Aboriginal and Torres Strait Islander research group at the National Acoustic Laboratories, and are ensuring that the co-design of recruitment is led by ANCHOR investigator (author KK) who is an otolaryngologist, hearing health specialist, and Worimi (First Nations) person. We are engaging with the Wukul Yabang Panel in Newcastle to facilitate collaboration with local Aboriginal and Torres Strait Islander community members to receive feedback on their research proposals. We anticipate contacting communities in at least three states/territories to ensure diversity of views. In parallel, the National Acoustic Laboratories HearOut Study is conducting yarning circles and interviews [34, 35] and an online survey with Aboriginal and Torres Strait Islander families and with community-based healthcare and education professionals to determine what outcomes matter to them in relation to persistent otitis media-related deafness and ear health care [36]. Both ANCHOR and HearOut Project teams are committed to conducting research in line with the NHMRC’s *Ethical conduct in research with Aboriginal and Torres Strait Islander peoples and communities* (spirit and integrity, cultural continuity, equity, reciprocity, respect, and responsibility), the principles of the Australian Institute of Aboriginal and Torres Strait Islander Code of Ethics for Aboriginal and Torres Strait Islander Research framework (1. Indigenous self-determination; 2. Indigenous leadership; 3. Impact and value; 4. Sustainability and accountability) and all other relevant Aboriginal and Torres Strait Islander health and medical Human Research and Ethics Committee (HREC) guidelines including the Collective benefit, Authority to control, Responsibility, and Ethics (CARE) Principles for Indigenous Data Governance [37].

Focus group and interview data are being transcribed verbatim, with accredited Auslan-English interpreters used where needed to produce written English transcripts. Analysis is ongoing: after each session, interviewers meet to review emerging themes and assess thematic saturation, at which point recruitment will cease. We are using inductive content analysis to identify content categories [38]. Content analysis allows for replicable and valid inferences to be made from data to garner knowledge, new insights, and inform practical action [38]. Two researchers are coding the transcripts; another researcher is independently coding a subset of the transcripts. The broader research team reviews the content categories and analysis to ensure consistency and quality assurance.

#### Step 2: Sorting outcomes into domains and ranking outcomes

Members of the Aim 3 Working Group, including members of the AusChildDeafness-CAG, are reviewing all Step 1 outcomes, and grouping them into ‘core outcome domains’. Outcomes are listed under these ‘core outcome domains’ to be ranked in two rounds of e-Delphi surveys and workshops, offered in both Auslan and English.

#### e-Delphi round one methodology

We are inviting child hearing health professionals and people with lived experience from across Australia to participate in the e-Delphi process. Recruitment will use the same strategies described for Aim 1, supplemented by email invitations through ANCHOR stakeholders, ANCHOR Investigator Team, and a co-developed recruitment video with the AusChildDeafness-CAG distributed via social media. The email contains a link to the online REDCap survey. The eDelphi survey and recruitment materials are developed in both Auslan and English.

Inclusion criteria for the e-Delphi Round One are: a young person who is deaf or hard of hearing (aged between 16 and 26 years); a parent/carer of a child or young person who is deaf or hard of hearing (aged 0 to 26 years); a deaf adult who uses Australian Sign Language (Auslan); a health professional, early intervention or allied health worker, support or advocacy professional or educator who has worked with at least 10 children or young people who are deaf or hard of hearing in the last 12 months; a researcher who has undertaken research into child hearing in the last 5 years; or a conference delegate attending the 2025 Australasian Newborn Hearing Screening Conference in Canberra.

Once consent to participate in the survey is provided, participants select the lived experience or professional group that they most identify with, and select the domains to rank. Participants rank each outcome according to a 9-point Likert scale (‘1’ = ‘not important’; ‘9’ = ‘critically important’). We are analysing the results from the e-Delphi Round One by the following sub-groups to ensure that the voices of minority groups are included:

Sub-group 1:

- The deaf community (those who identify as deaf and primarily use Auslan as their primary communication mode).
- Professionals who use Auslan fluently and/or identify as deaf or hard of hearing, and.
- Parent/carers of a child whose primary communication mode is Auslan.

Sub-group 2:

- Hearing health professionals,
- Parents/carers of DHH children whose primary mode of communication is speech, and.
- Young people who are DHH whose primary mode of communication is speech.

To determine which outcomes progress through to the e-Delphi Round Two survey versus to workshops for further ranking, the following threshold criteria are used:

- Outcomes are included in the e-Delphi Round Two when ranked as ‘critically important’ (ranked 8 or 9) by > 80% of participants AND ‘unimportant’ (ranked 1, 2 or 3) by < 15% of participants in either of the two sub-groups.
- Outcomes do not progress to e-Delphi Round Two or workshops when they are scored as ‘critically important’ (ranked 8 or 9) by less than 50% of participants overall.
- Outcomes that do not fall into either of the two categories above (‘uncertain outcomes’) are taken to the workshops for further ranking.

#### Workshops methodology

The aim of the workshops, an additional step in between the two rounds of e-Delphi, is to further rank the list of ‘uncertain outcomes’ in a group setting to allow engagement, discussions and reflections. This additional step is in anticipation of the extensive list of outcomes from e-Delphi Round One that require further refinement, in view of the vast array of different stakeholders involved in the ranking process. Three workshops are held, tailoring to a specific group: 1) Parents/carers of children who are DHH; 2) Child hearing health professionals; and 3) Parents/carers and young people whose primary mode of communication is Auslan (deaf community).

Each interactive workshop uses a resource allocation methodology to rank the ‘uncertain outcomes’. The ‘uncertain outcomes’ from each core outcome domain from e-Delphi Round One are listed on posters. Each participant is given a restricted number of coloured green versus red stickers to put against the ‘intermediate outcomes’ on the posters. Green stickers stand for the ‘most important outcomes’ and red stickers stand for the ‘least important’ outcomes. For each core outcome domain, participants are given the number of green vs red stickers corresponding to 1/3 of the number of outcomes per domain. For example, if a domain has 12 outcomes, participants have four green stickers and four red stickers available to rank against each outcome.

**Fig. 4:**
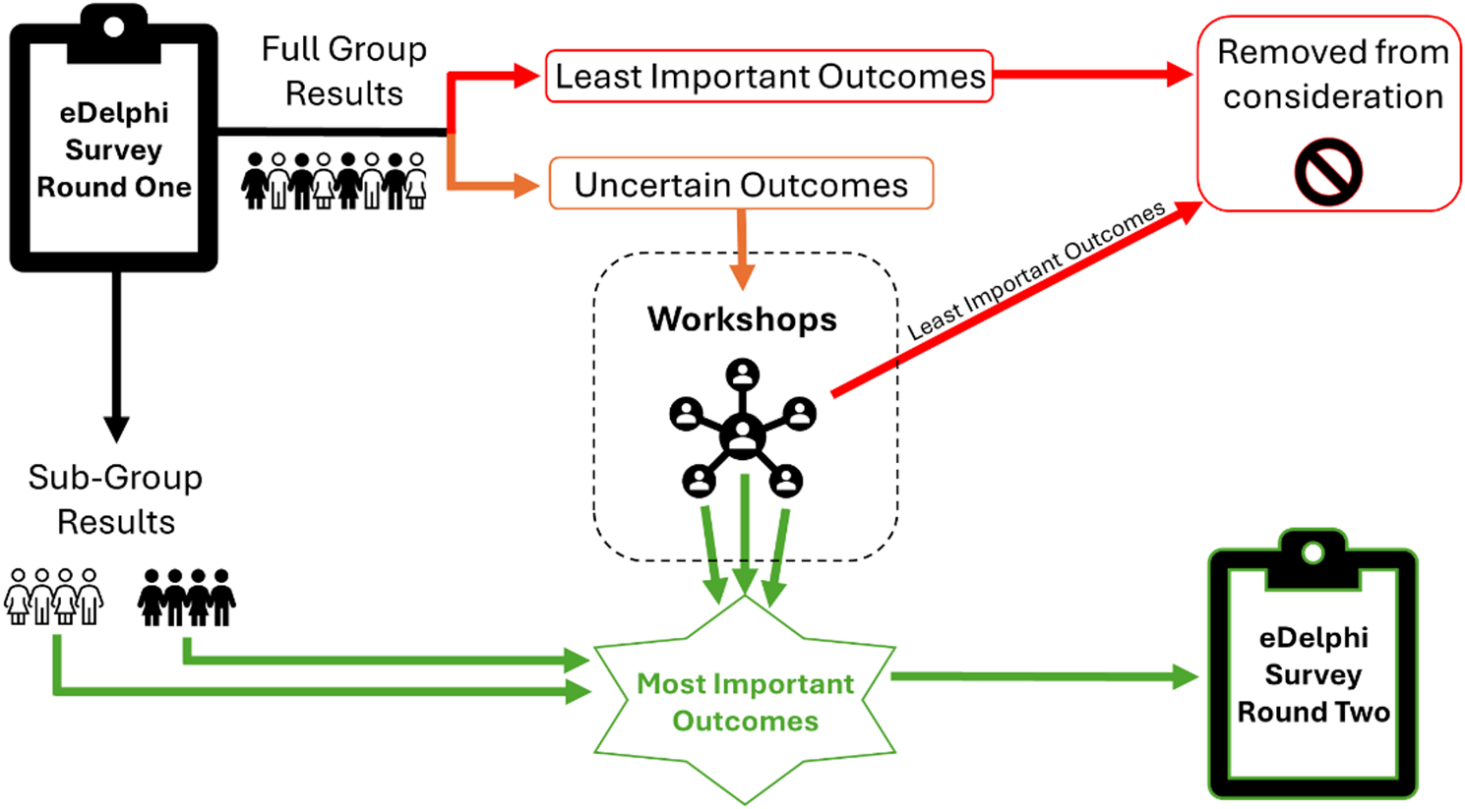
A flow-chart to represent which outcomes progress through to e-Delphi survey round two

Results from the workshops are analysed by counting the green and red stickers for each of the outcomes for each core outcome domain. Outcomes progress through to e-Delphi Round Two if they are scored as ‘most important’ (green stickers) by greater than 50% of participants in each of the three workshops. Figure 4 illustrates how outcomes are selected to progress from e-Delphi Round One to eDelphi Round Two.

#### e-Delphi round two and endorsement meeting methodology

We re-invite participants of the e-Delphi Round One and workshop participants to participate in e-Delphi Round Two, through an online REDCap survey available in both Auslan and English. Similar to the e-Delphi Round One, participants rank each outcome according to a 9-point Likert scale, with ‘1’ representing ‘not important’ and ‘9’ representing ‘critically important’. We also ask participants to express how many outcomes they view are acceptable or feasible to collect for each child who is DHH for each service they are engaged in.

**Fig. 5:**
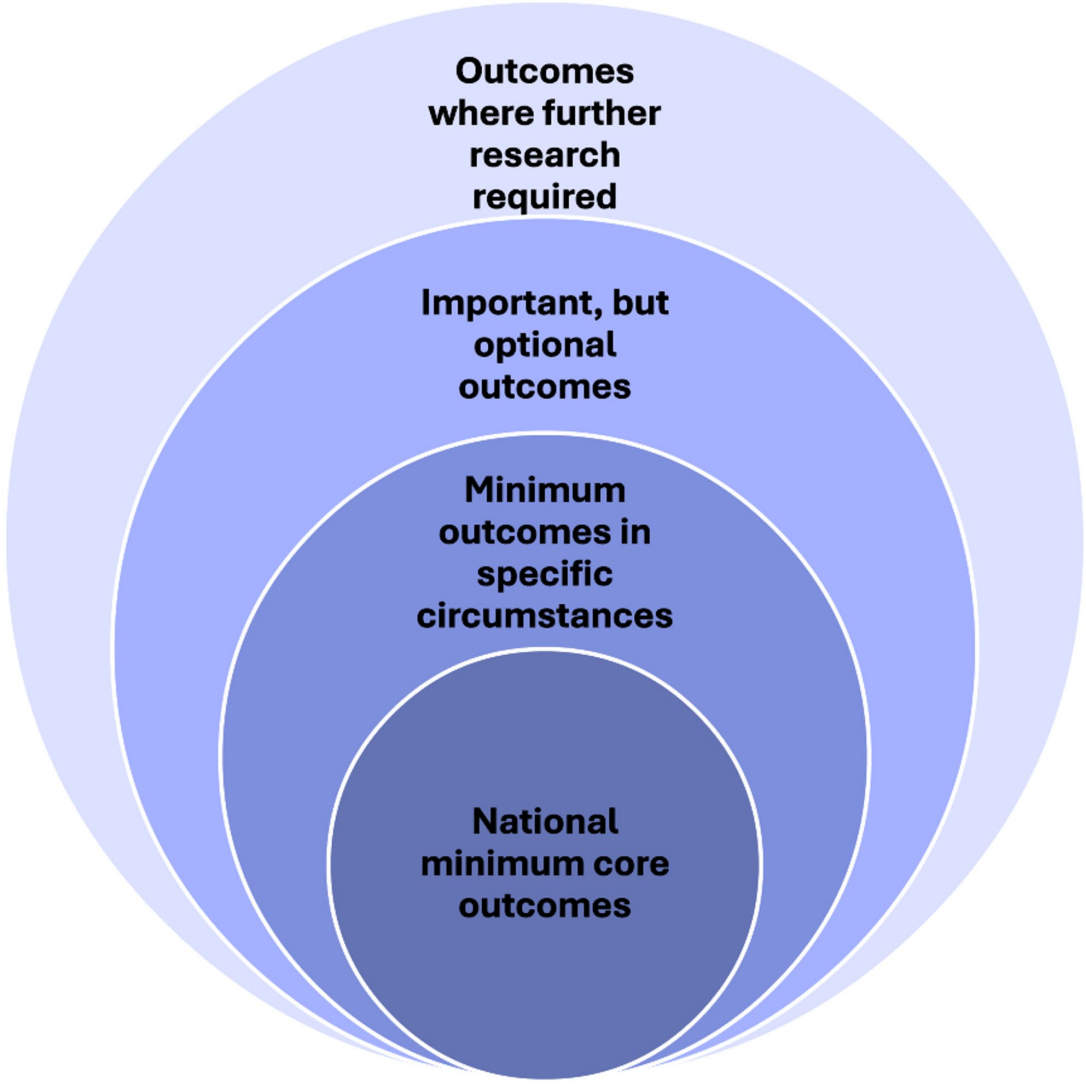
Representation of the “onion layers” which will be used for grouping outcomes. Adapted from OMERACT

Outcomes are included in the ‘Endorsement Meeting’ when ranked as ‘critically important’ (ranked 8 or 9) by > 80% of participants AND ‘unimportant’ (ranked 1, 2 or 3) by < 15% of participants. The ANCHOR Investigator Team, the AusChildDeafness-CAG, and representatives from the deaf community will ratify the results and group them into “onion layers” (see Fig. 5) [39]. The final number of outcomes included in the COS will be guided by the responses from e-Delphi participants regarding how many outcomes they view are acceptable or feasible to collect for each child. It is also anticipated that further future work will be required to determine the tools and measures for each COS.

### Timeline of ANCHOR

**Fig. 6:**
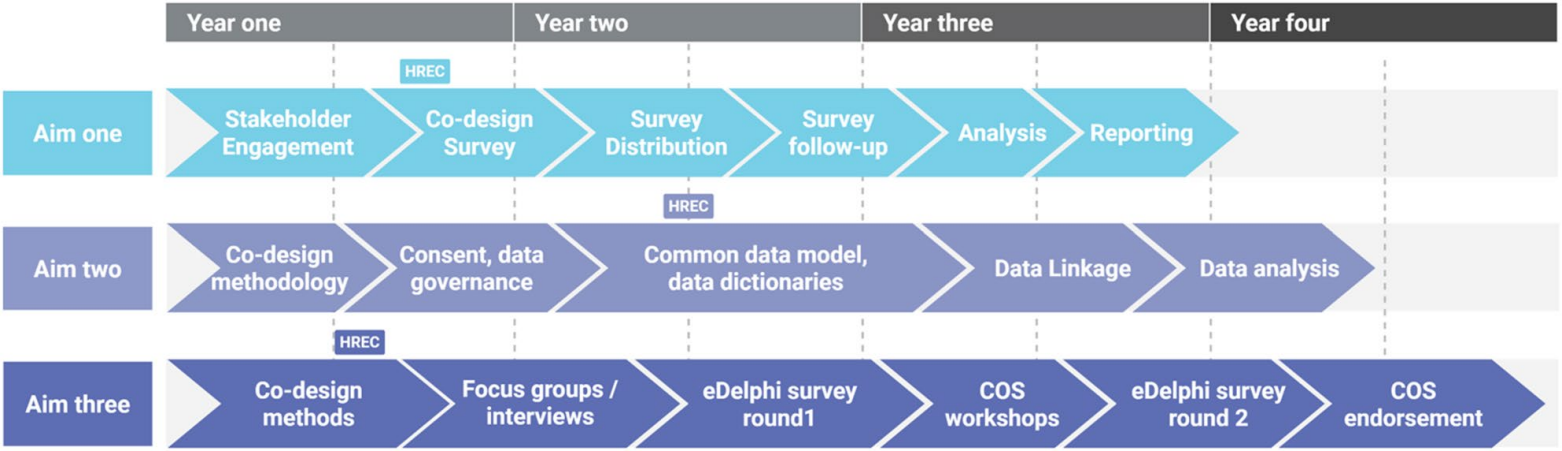
Timeline of the ANCHOR program

The proposed timeline of ANCHOR activities is shown in Fig. 6.

## Discussion

ANCHOR will be a world-first Learning Health System to sustainably and systematically track the outcomes of DHH children at a national level. ANCHOR’s methodology, using community engagement at its heart, will also serve as a prototype for other health conditions using data linkages to sustainably capture large-scale data, to ensure data typically missing from vulnerable families who do not participate in traditional research can be included. ANCHOR builds upon VicCHILD that collects outcomes on children identified by UNHS in Victoria [28] and the Queensland QChild database that tracks program-level outcomes from UNHS to intervention. Currently, few child deafness registries exist internationally, and none, to our knowledge, use data linkages of existing datasets. Moreover, few registries effectively identify and track vulnerable populations in a meaningful, forward-looking manner to address the disparity in child deafness outcomes. The German Registry for Hearing Loss in Children reports on early diagnosis and therapy [40], the Sing Registry reports on genetic causes of hearing loss [41], the Listening and Spoken Language Data Repository reports on functional communication outcomes [42]. Norway recently established a national registry for childhood hearing loss [43], similar to the Swedish national registry [44].

Large-scale child deafness registries are critical tools to better understand the factors that contribute to the variations in developmental outcomes — both within the population of DHH children and between that population and their typically hearing peers [10]. ANCHOR’s whole of population data can address service access and research questions prospectively and continuously rather than requiring expensive individually planned and executed studies requiring once-off data collections. ANCHOR will provide the necessary data to confirm the results from a recent economic analysis of the Australian UNHS that suggested a modest cost-benefit: an incremental cost-effectiveness ratio of $48,000 per QALY gained per diagnosed child from UNHS compared with targeted screening [5]. In addition, ANCHOR could provide evidence to support a 2022 report that estimated optimising access to Auslan language could provide an economic benefit of $27 million annually to the Australian economy [45].

In setting up a national data system like ANCHOR, we anticipate there will be numerous challenges to overcome. To enable successful data linkage, we need to ensure services and organisations collect the same or similar outcome measures, with agreed definitions, as a minimum standard. This could be facilitated by developing a national COS. Most published works on developing a COS for deafness has been in the adult deafness sector [46–48]. For DHH children, there are published COS for otitis media with effusion and for children with cleft palate [49, 50], but not yet for sensorineural hearing loss. Our work along with that from the PONCHO study will address this. In developing a national COS, we are putting community and stakeholder voices at the core of the work, because a COS that is meaningful to DHH children and their communities will help them make informed decisions about engaging with different support pathways and interventions. Moreover, professionals and service providers would be more likely to engage with collecting the appropriate outcomes information if the COS matters to them. However, often the outcomes that matter to professionals – whether they be in medicine, allied health, or education – can be very different from those that may be perceived to be important to DHH children and their communities [46]. We anticipate overcoming differences in perspectives by respectfully engaging and partnering with the many child deafness communities and stakeholders, and including perspectives from all stakeholders equally through the Aim 3 processes, noting the voices of families with lived experiences and minority groups, such as the deaf and Aboriginal and Torres Strait Islander communities. At the end of the funded three-year program, we expect to establish several subsets of COS domains, with further funding required to determine the measures to be used in the future.

Navigating data linkage in Australia is inherently complex due to the nation’s federal structure, in which data custodians across multiple jurisdictions (federal, state, and territory) independently govern various aspects of data collection, privacy, and health service delivery. This fragmentation often results in extended timelines for negotiating agreements between jurisdictions and agencies. To streamline this process, we have leveraged national infrastructure of the Population Health Research Network (PHRN) and its established relationships with State and Commonwealth linkage agencies, enabling us to progress data linkage as efficiently as possible.

In negotiating data linkage with stakeholders, we have encountered some organisational resistance driven by ethical, legal, data privacy, and security concerns. An additional challenge lies in the considerable variation in how data are collected across existing services and organisations. These differences include variability in patient and client consent processes, data quality and consistency, documentation standards or metadata, and technical formats for data storage. By collaborating closely with key stakeholders to address ethical, technical and operational barriers, we intend to overcome the logistical complexities inherent data linkage and ensure the successful delivery of the Aim 2 program of work.

## Conclusion

A comprehensive, person-centred, outcomes-focused data system will generate robust and actionable evidence to drive effective care, advance knowledge, and improve outcomes for DHH children. Australia has shown extraordinary global leadership in world class technologies for DHH children. However, without an equally high-quality integrated data systems, shaped by the priorities of families and young people themselves, many DHH children will continue to experience poorer outcomes than their hearing peers. Stakeholders will also lack understanding of what “best life outcomes” entail and how to measure them. ANCHOR unites researchers, families, and stakeholders who share the expertise and commitment needed to develop these critical systems. Building on existing successful data resources, it will create a two-state prototype for a national child hearing and deafness data system, one of the first of its kind worldwide. Ultimately, it will give DHH children the best opportunities to reach their full potential and live happy, fulfilled lives.

## Supplementary Information

Below is the link to the electronic supplementary material.

Supplementary Material 1

Supplementary Material 2

Supplementary Material 3

## Data Availability

Not applicable as this a study protocol.

## Abbreviations

Auslan: Australian Sign Language
AusChildDeafness-CAG: Australian Childhood Deafness Research Community Advisory Group
AIHW: Australian Institute of Health and Welfare
ANCHOR: Australian National Child Hearing Health Outcomes Registry
COS: Core Outcome Set
COMET: Core Outcome Measures in Effectiveness Trials
CVDL: Centre for Victorian Data Linkage
DLQ: Data linkage Queensland
DHH: Deaf and/or hard of hearing (The lower case ‘d’ (‘deaf’) is used as an all-encompassing term to acknowledge different identities with which a deaf or hard of hearing person may identify and the different means by which they may communicate, including Australian Sign Language, Auslan)
EVSI: Expected Value of Sample Information
HREC: Human Research Ethics Committee
ID: Identification number
LOCHI: Longitudinal Outcomes of Children with Hearing Impairment
PHRN: Population Health Research Network
PII: Personal identifying information
PONCHO: Prioritising Outcomes iN Childhood Hearing lOss
SURE: Secure Unified Research Environment
UNHS: Universal newborn hearing screening
VALT: Victorian data Access Linkage Trust
VicCHILD: Victorian Childhood Hearing Longitudinal Databank

## References

[1] Wake M, Ching TY, Wirth K, Poulakis Z, Mensah FK, Gold L, et al. Population outcomes of three approaches to detection of congenital hearing loss. Pediatrics. 2016;137(1):1–10. 10.1542/peds.2015-1722. 10.1542/peds.2015-1722 PMC4702017 26704085

[2] Ching TYC, Dillon H, Button L, Seeto M, Van Buynder P, Marnane V, et al. Age at intervention for permanent hearing loss and 5-year language outcomes. Pediatrics. 2017;140(3). 10.1542/peds.2016-4274. 10.1542/peds.2016-4274 PMC5574730 28864712

[3] Australian national hearing screening committee, editor australian consensus statement on universal neonatal hearing screening. Universal neonatal hearing screening in Australia. A national forum for consensus and implementation Adelaide, Australia; 2001.

[4] Australian government department of health and aged care. National framework for neonatal hearing screening Australia August 2013.

[5] Sharma R, Gu Y, Sinha K, Ching T, Marnane V, Gold L, et al. An economic evaluation of australia’s newborn hearing screening program: A within-study cost-effectiveness analysis. Ear Hear. 2021;43(3):972–83. 10.1097/AUD.0000000000001153. 10.1097/AUD.0000000000001153 PMC9275830 34772837

[6] Ching TYC, Oong R, van Wanrooy E. The ages of intervention in regions with and without universal newborn hearing screening and prevalence of childhood Heaering impairment in Australia. Australian New Z J Audiol. 2006;28(2):137–50. 10.1375/audi.28.2.137.

[7] U.S. Centres for Disease control and prevention. early hearing detection and intervention 1-3-6 Benchmarks 2024. Available from: https://www.cdc.gov/hearing-loss-children/articles/baby-hearing-screening-infographic.html.

[8] Australian National Joint Committee on Infant Hearing. Position statement: principles and guidelines for early hearing detection and intervention programs. J Early Hear Detect Intervention. 2019;4(2):1–44. 10.15142/fptk-b748.

[9] Gray JM. New concepts in screening. Br J Gen Pract. 2004;54(501):292. 15113498 PMC1314856

[10] Carew P, Mensah F, Rance G, Flynn T, Poulakis Z, Wake M. Mild–moderate congenital hearing loss: secular trends in outcomes across four systems of detection. Child Care Health Dev. 2018;44(1):71–82. 10.1111/cch.12477. 28612343 10.1111/cch.12477

[11] Carew P, Shepherd DA, Smith L, Soh QR, Sung V. Language and health-related quality of life outcomes of children early-detected with unilateral and mild bilateral hearing loss. Front Pead. 2023;11:1210282. 10.3389/fped.2023.1210282. 10.3389/fped.2023.1210282 PMC10461396 37645035

[12] Carew P, Shepherd DA, Smith L, Howell T, Lin M, Bavin EL, et al. Spoken expressive vocabulary in 2-Year-Old children with hearing loss: A community study. Children. 2023;10(7):1223. 10.3390/children10071223. 37508720 10.3390/children10071223 PMC10377817

[13] Ching TY, Dillon H, Button L, Seeto M, Van Buynder P, Marnane V, et al. Age at intervention for permanent hearing loss and 5-year Language outcomes. Pediatrics. 2017;140(3). 10.1542/peds.2016-4274. 10.1542/peds.2016-4274 PMC5574730 28864712

[14] Burns JF, Thomson NJ. Review of ear health and hearing among Indigenous Australians Perth: Australian Indigenous HealthInfoNet; 2013.

[15] Wolff R, Hommerich J, Riemsma R, Antes G, Lange S, Kleijnen J. Hearing screening in newborns: systematic review of accuracy, effectiveness, and effects of interventions after screening. Arch Dis Child. 2009;95(2):130–5. 10.1136/adc.2008.151092. 19329444 10.1136/adc.2008.151092

[16] Nelson EC, Dixon-Woods M, Batalden PB, Homa K, Van Citters AD, Morgan TS, et al. Patient focused registries can improve health, care, and science. BMJ. 2016;354:i3319. 10.1136/bmj.i3319. 27370543 10.1136/bmj.i3319 PMC5367618

[17] Callander EJ, Fox H. What are the costs associated with child and maternal healthcare within Australia? A study protocol for the use of data linkage to identify health service use, and health system and patient costs. BMJ Open. 2018;8(2):e017816. 10.1136/bmjopen-2017-017816. 29437751 10.1136/bmjopen-2017-017816 PMC5829863

[18] Panteli D, Polin K, Webb E, Allin S, Barnes A, Degelsegger-Márquez A, et al. Health and care data: approaches to data linkage for evidence-informed policy. World Health Organization, Regional office for Europe; 2023. 37489953

[19] Wake M, Hu YJ, Warren H, Danchin M, Fahey M, Orsini F, et al. Integrating trials into a whole-population cohort of children and parents: statement of intent (trials) for the generation Victoria (GenV) cohort. BMC Med Res Methodol. 2020;20:1–15. 10.1186/s12874-020-01111-x. 10.1186/s12874-020-01111-x PMC7512047 32972373

[20] Johnston T. The linkage landscape: what data is being linked and how it is being used. Queensland Health Data Linkage Symposium; Queensland Statistical Analysis and Linkage Unit, Queensland Health; 2023 Nov 16.

[21] Williamson PR, Altman DG, Bagley H, Barnes KL, Blazeby JM, Brookes ST, et al. The COMET handbook: version 1.0. Trials. 2017;18(3):280. 10.1186/s13063-017-1978-4. 28681707 10.1186/s13063-017-1978-4 PMC5499094

[22] Victorian Comprehensive Cancer Centre. Model of Consumer Engagement 2022.

[23] Fleckenstein M, Obaidi A, Tryfona NA. A review of data valuation approaches and building and scoring a data valuation model. review of data valuation approaches and building and scoring a data valuation model. Harv Data Sci Rev. 2023;5(1). 10.1162/99608f92.c18db966.

[24] Soekhai V, de Bekker-Grob EW, Ellis AR, Vass CM. Discrete choice experiments in health economics: past, present and future. PharmacoEconomics. 2019;37(2):201–26. 10.1007/s40273-018-0734-2. 30392040 10.1007/s40273-018-0734-2 PMC6386055

[25] Gumbie M, Parkinson B, Dillon H, Bowman R, Song R, Cutler H. Cost-Effectiveness of screening preschool children for hearing loss in Australia. Ear Hear. 2021;43(3):1067–78. 10.1097/aud.0000000000001134. 10.1097/AUD.0000000000001134 34753856

[26] Strong M, Oakley JE, Brennan A, Breeze P. Estimating the expected value of sample information using the probabilistic sensitivity analysis sample: a fast, nonparametric regression-based method. Med Decis Making. 2015;35(5):570–83. 10.1177/0272989X15575286. 25810269 10.1177/0272989X15575286 PMC4471064

[27] Edney LC, Haji Ali Afzali H, Cheng TC, Karnon J. Estimating the reference incremental Cost-Effectiveness ratio for the Australian health system. PharmacoEconomics. 2018;36(2):239–52. 10.1007/s40273-017-0585-2. 29273843 10.1007/s40273-017-0585-2

[28] Sung V, Smith L, Poulakis Z, Burt RA, Carew P, Tobin S, et al. Data resource profile: the Victorian childhood hearing impairment longitudinal databank (VicCHILD). Int J Epidemiol. 2019;48(5):1409–10. 10.1093/ije/dyz168. h. 31411681 10.1093/ije/dyz168

[29] Wilkinson MD, Dumontier M, Aalbersberg IJ, Appleton G, Axton M, Baak A, et al. The FAIR Guiding Principles for scientific data management and stewardship. Sci Data. 2016;3(1):160018. 10.1038/sdata.2016.18. 10.1038/sdata.2016.18 PMC4792175 26978244

[30] Williamson PR, Altman DG, Bagley H, Barnes KL, Blazeby JM, Brookes ST, et al. The COMET handbook: version 1.0. Trials. 2017;18(3):1–50. 10.1186/s13063-017-1978-4. 28681707 10.1186/s13063-017-1978-4 PMC5499094

[31] Bruce I, Morris R, O’Malley L, Lin Y, O’Driscoll M, Booth R et al. COMET Initiative: Prioritising Outcomes in Childhood Hearing loss (The PONCHO study). 2021. Available from: https://www.comet-initiative.org/studies/details/1362.

[32] Harris PA, Taylor R, Minor BL, Elliott V, Fernandez M, O’Neal L, et al. The REDCap consortium: Building an international community of software platform partners. J Biomed Inf. 2019;95:103208. 10.1016/j.jbi.2019.103208. 10.1016/j.jbi.2019.103208 PMC7254481 31078660

[33] Harris PA, Taylor R, Thielke R, Payne J, Gonzalez N, Conde JG. Research electronic data capture (REDCap)--a metadata-driven methodology and workflow process for providing translational research informatics support. J Biomed Inf. 2009;42(2):377–81. 10.1016/j.jbi.2008.08.010. 10.1016/j.jbi.2008.08.010 PMC2700030 18929686

[34] Bessarab D, Ng’Andu B. Yarning about yarning as a legitimate method in Indigenous research. Int J Crit Indigenous Stud. 2010;3(1):37–50. 10.5204/ijcis.v3i1.57.

[35] Kennedy M, Maddox R, Booth K, Maidment S, Chamberlain C, Bessarab D. Decolonising qualitative research with respectful, reciprocal, and responsible research practice: a narrative review of the application of yarning method in qualitative aboriginal and Torres Strait Islander health research. Int J Equity Health. 2022;21(1):134. 10.1186/s12939-022-01738-w. 36100899 10.1186/s12939-022-01738-w PMC9472448

[36] O’Keeffe I, Nash J, Harkus S, Ward M, Austin L, Hay E, et al. Early results from hearout (Hearing Health Outcomes for Aboriginal and Torres Strait Islander children)Newcastle: OMOZ Newcastle; 2024.

[37] Australian research data commons. care principles: National research infrastcuture for Australia. 2024 Available from: https://ardc.edu.au/resource/the-care-principles/.

[38] Vears DF, Gillam L. Inductive content analysis: A guide for beginning qualitative researchers. Focus Health Prof Education: Multi-disciplinary J. 2022;23(1):111–27. 10.11157/fohpe.v23i1.544.

[39] Maxwell LJ, Beaton DE, Shea BJ, Wells GA, Boers M, Grosskleg S, et al. Core domain set selection according to OMERACT filter 2.1: the OMERACT methodology. J Rhuematol. 2019;46(8):1014–20. 10.3899/jrheum.181097. 10.3899/jrheum.181097 30770502

[40] Finckh-Krämer U, Spormann-Lagodzinski M, Gross M. German registry for hearing loss in children: results after 4 years. Int J Pediatr Otorhinolaryngol. 2000;56(2):113–27. 10.1016/s0165-5876(00)00401-8. 11115684 10.1016/s0165-5876(00)00401-8

[41] The Sing Registry: The Genetic Sensorineural Hearing Loss Registry: Akouos. 2021 Available from: https://singregistry.com/#about.

[42] Bradham TS, Fonnesbeck C, Toll A, Hecht BF. The Listening and Spoken Language Data Repository: Design and Project Overview. Lang Speech Hear Serv Sch. 2018;49(1):108–20. 10.1044/2017_lshss-16-0087. 10.1044/2017_LSHSS-16-0087 PMC6105089 29222559

[43] Mattsson TS, Nilsen AH, Wennberg S. Establishment of the Norwegian hearing register for children. Front Hum Neurosci. 2024;18:1400005. 10.3389/fnhum.2024.1400005. 39135757 10.3389/fnhum.2024.1400005 PMC11317270

[44] Konradsson K, Jarvholm M. Introducing a National paediatric hearing register in Sweden. Audiol Med. 2004;2(2):113–22. 10.1080/16513860410031984.

[45] D’Rosario M, Dawson E, Our Culture. Our Value: The social and economic benefits of Auslan, a research report by per capita for deaf Australia and deaf connect 2022.

[46] Allen D, Hickson L, Ferguson M. Defining a patient-centred core outcome domain set for the assessment of hearing rehabilitation with clients and professionals. Front NeuroSci. 2022;16:787607. 10.3389/fnins.2022.787607. 35592258 10.3389/fnins.2022.787607 PMC9110701

[47] Hill-Feltham PR, Johansson ML, Hodgetts WE, Ostevik AV, McKinnon BJ, Monksfield P, et al. Hearing outcome measures for conductive and mixed hearing loss treatment in adults: a scoping review. Int J Audiol. 2021;60(4):239–45. 10.1080/14992027.2020.1820087. 32985284 10.1080/14992027.2020.1820087

[48] Katiri R, Hall DA, Buggy N, Hogan N, Van de Horobin A, et al. Core rehabilitation outcome set for single sided deafness (CROSSSD) study: protocol for an international consensus on outcome measures for single sided deafness interventions using a modified Delphi survey. Trials. 2020;21(1):238. 10.1186/s13063-020-4094-9. 32131880 10.1186/s13063-020-4094-9 PMC7057560

[49] Harman NL, Bruce IA, Callery P, Tierney S, Sharif MO, O’Brien K, et al. MOMENT–Management of otitis media with effusion in cleft palate: protocol for a systematic review of the literature and identification of a core outcome set using a Delphi survey. Trials. 2013;14:1–8. 10.1186/1745-6215-14-70. 23497540 10.1186/1745-6215-14-70 PMC3716725

[50] Liu PZ, Ismail-Koch H, Stephenson K, Donne AJ, Fergie N, Derry J, et al. A core outcome set for research on the management of otitis media with effusion in otherwise-healthy children. Int J Pediatr Otorhinolaryngol. 2020;134:110029. 10.1016/j.ijporl.2020.110029. 32272377 10.1016/j.ijporl.2020.110029

