# Supplementary material for: Study protocol - the Australian National Child hearing Health Outcomes Registry (ANCHOR): collecting and connecting national data into a child deafness Learning Health System
Source: BMC Health Serv Res. 2026 Mar 4;26:502. doi: 10.1186/s12913-026-14123-y (PMC13067657; doi:10.1186/s12913-026-14123-y)
Supplement: Supplementary file 1 — Supplementary Material 1 [file 12913_2026_14123_MOESM1_ESM.pdf]

Study Protocol - The Australian National Child Hearing Health Outcomes Registry (ANCHOR): Collecting and connecting national data into a child deafness Learning Health System

Additional File 1

Figure 1: Existing mature services and datasets that will contribute to ANCHOR's vision.

Possible Core Outcome Domains:

hearing diagnostics; middle ear status; hearing devices; communication; quality of life & wellbeing; development; education; medical; support/services access

		hearing diagnostics	middle ear status	hearing devices	communication	medical	quality of life & wellbeing	development	education	support/services access
Services available in most states in Australia	Universal Newborn Hearing Screening	•								•
	Diagnostic Audiology	•	•							
	Hearing Australia	•	•	•	•					
	Cochlear implant and specialised paediatric clinics	•	•	•	•	•		•		•
	Parent mentoring<br>Parent support									•
	Early intervention			•	•			•		
	School								•	•
	National Disability Insurance Scheme (NDIS)	•		•	•	•	•	•		•
	Maternal child health	•			•	•	•	•		•
	Aboriginal and Torres Strait Islander Ear Health Check	•	•							
VIC	Generation Victoria	•				•	•	•	•	
	Victorian Childhood Hearing Longitudinal Databank	•	•	•	•	•	•	•	•	
QLD	Healthy Hearing Program Database<br>QChild	•	•	•	•	•		•		•
