# Supplementary material for: Study protocol - the Australian National Child hearing Health Outcomes Registry (ANCHOR): collecting and connecting national data into a child deafness Learning Health System
Source: BMC Health Serv Res. 2026 Mar 4;26:502. doi: 10.1186/s12913-026-14123-y (PMC13067657; doi:10.1186/s12913-026-14123-y)
Supplement: Supplementary file 2 — Supplementary Material 2 [file 12913_2026_14123_MOESM2_ESM.pdf]

Study Protocol - The Australian National Child Hearing Health Outcomes Registry (ANCHOR): Collecting and connecting national data into a child deafness Learning Health System

Additional File 2

Table 1: Glossary

Term	Definition
Medicare	Medicare is Australia's universal health insurance scheme. Medicare pays for some or all of the costs of various medical services, including services delivered in public and private hospitals. It ensures all Australians have equitable access to health care when they need it, regardless of where they live or their ability to pay. For more information: https://www.health.gov.au/topics/medicare?language=und
National Disability Insurance Scheme (NDIS)	The National Disability Insurance Scheme (NDIS) provides support to eligible people with disability, their families and carers. It is jointly governed and funded by the Australian Government, and participating state and territory governments. The National Disability Insurance Agency (NDIA) helps to implement the NDIS by providing information and referrals, empowering people with diabsility and providing service providers with transparency. For more information: https://www.ndis.gov.au/
National Health and Medical Research Council (NHMRC)	The National Health and Medical Research Council (NHMRC) is the main statutory authority of the Australian Government responsible for medical research. As an independent arm of the Department of Health, the NHMRC funds high quality health and medical research, builds research capability in Australia, supports the translation of health and medical research into better health outcomes, and promote the ethics and integrity in research. For more information: https://www.nhmrc.gov.au/
Australian Institute of Health and Welfare (AIHW)	The AIHW is an independent statutory Australian Government agency producing authoritative and accessible health and welfare statistics. AIHW aims to improve the health and welfare of all Australians by making available information and statistics that can help shape and improve the health of our community through better services and programs. AIHW provides data services including data integration. For more information: https://www.aihw.gov.au/
Data Integrating Authority	Integrating Authorities are agencies ultimately accountable for the implementation of data integration or linkage projects. Accredited Integrating Authorities are assessed by the Australian Government Oversight Board as having the infrastructure and capability to undertake high risk data integration projects by meeting a set of criteria agreed by the Commonwealth Portfolio Secretaries, including being subject to relevant privacy legislation.

	For more information: https://toolkit.data.gov.au/data-integration/roles-and-responsibilities/integrating-authorities.html
Secure Unified Access Environment (SURE)	SURE (Secure Unified Research Environment) is a secure platform for the sharing and analysis of sensitive health and other data. SURE offers custodians a flexible and trusted way to share health data with the research community, while also giving researchers access to approved linked data.<br>For more information: https://www.saxinstitute.org.au/solutions/sure/
Centre for Victorian Data Linkage (CVDL)	The Centre for Victorian Data Linkage (CVDL) is part of the Victorian state government Agency for Health Information (VAHI), which is Victoria's end-to-end health data agency.<br>The CVDL are Victoria's specialist data linkage unit and provide a range of services to researchers and other Victorian Government departments. The CVDL maintains the Victorian Linkage Map (VLM) and Integrated Data Resource (IDR), a collection of almost 40 datasets which are linked on a monthly basis.<br>For more information: https://vahi.vic.gov.au/ourwork/data-linkage
Victorian data Access Linkage Trust (VALT)	The Victorian data Access Linkage Trust (VALT) is a secure online platform developed by the Centre for Victorian Data Linkage (CVDL) for release and analysis of linked, de-identified data for approved projects.<br>For more information: https://vahi.vic.gov.au/ourwork/data-linkage/valt-information-and-faq
Data Linkage Queensland (DLQ)	The Statistical Analysis and Linkage Unit within the Statistical Services Branch of Queensland Health is also known as Data Linkage Queensland (DLQ). DLQ routinely links many Departmental and other health-related datasets, and performs data linkage to facilitate and promote health service planning, management, monitoring and evaluation, and research within Queensland. Additionally, DLQ is responsible for much of Queensland's health data linkage for Commonwealth requirements, national committees, and cross-jurisdictional research projects.<br>For more information: https://www.health.qld.gov.au/hsu/link/datalink

List of Stakeholders from Figure 1 and Figure 4 and their websites for further information

Name of Organisation	Link to website

Audiology Australia	https://audiology.asn.au/
Aurora School	https://www.auroraschool.vic.edu.au/
Australian Institute of Health and Welfare (AIHW)	https://www.aihw.gov.au/
Australasian Newborn Hearing Screening Committee	https://www.newbornhearingscreening.com.au/
Aussie Deaf Kids	https://www.aussiedeafkids.org.au/
Children's Health Queensland	https://www.childrens.health.qld.gov.au/
Childhood Hearing Australasia Medical Professionals (CHAMP) Network	https://www.newbornhearing screening.com.au/champ-network/
Clinical Epidemiology and Biostatistics Unit (CEBU), MCRI	https://www.mcri.edu.au/research/research-areas/population-health/clinical-epidemiology-biostatistics-cebu
Deaf Australia	https://deafaustralia.org.au/
Deaf Children Australia	https://www.deafchildrenaustralia.org.au/
Deaf Connect	https://deafconnect.org.au/
Deafness Forum	https://www.deafnessforum.org.au/
Deaf Victoria	https://www.deafvictoria.org.au/
Dubbo and District Parent Support Group	https://www.hearourheart.org/dubbo--district-deaf-club.html
Generation Victoria (GenV)	https://www.genv.org.au/
Hearing Australia	https://www.hearing.com.au/
Hear and Say	https://www.hearandsay.org.au/
Hear for Kids	https://deafconnect.org.au/services/therapy-and-family-services/hear-for-kids-in-school
Macquarie University<br>Centre for the Health Economy	https://www.mq.edu.au/<br>https://www.mq.edu.au/research/research-centres-groups-and-facilities/prosperous-economies/centres/centre-for-the-health-economy
Municipal Association of Victoria	https://www.mav.asn.au/
Murdoch Children's Research Institute (MCRI)	https://www.mcri.edu.au/
National Association of Australian Teachers of the Deaf	https://naatd.com.au/
National Aboriginal Community Controlled Health Organisation (NACCHO)	https://www.naccho.org.au/
National Acoustic Laboratories	https://www.nal.gov.au/
National Disability Insurance Scheme	https://www.ndis.gov.au/
NextSense	https://www.nextsense.org.au/
New South Wales Department of Education Hear our Heart Ear Bus Project	https://www.hearourheart.org/
Parents of Deaf Children	https://www.podc.org.au/

Healthy Hearing Queensland	https://www.childrens.health.qld.gov.au/our-work/healthy-hearing
Queensland Audiology Working Group	Working group of Queensland audiologists
Rumbalara Aboriginal Co-operative	https://rumbalara.org.au/
Telethon Speech and Hearing	https://www.tsh.org.au/
The Royal Children's Hospital	https://www.rch.org.au/home/
The Royal Victorian Eye and Ear Hospital Cochlear Implant Clinic	https://eyeandear.org.au/patients-visitors/essential-patient-and-visitor-information/coming-for-a-specialist-appointment/specialist-clinics/cochlear-implant/
The Shepherd Centre	https://shepherdcentre.org.au/
Usher Kids Australia	https://usherkidsaustralia.com/
Victorian Aboriginal Community Controlled Health Organisation (VACCHO)	https://www.vaccho.org.au/
Victorian Childhood Hearing Longitudinal Databank (VicCHILD)	https://www.mcri.edu.au/research/projects/vicchild
Victorian College for the Deaf	https://www.vcd.vic.edu.au/
Victorian Department of Education and Training	https://www.vic.gov.au/education
Victorian Diagnostic Audiology Group (VDAG)	Working group of Victorian Audiologists
Victorian Infant Hearing Screening Program (VIHSP)	https://www.rch.org.au/vihsp/
Western Australia Foundation for Deaf Children	https://wafdc.org.au/
Yeerongpilly Early Childhood Development Program	https://yeerongpillyecdp.eq.edu.au/
