# Supplementary material for: Study protocol - the Australian National Child hearing Health Outcomes Registry (ANCHOR): collecting and connecting national data into a child deafness Learning Health System
Source: BMC Health Serv Res. 2026 Mar 4;26:502. doi: 10.1186/s12913-026-14123-y (PMC13067657; doi:10.1186/s12913-026-14123-y)
Supplement: Supplementary file 3 — Supplementary Material 3 [file 12913_2026_14123_MOESM3_ESM.pdf]

ANCHOR Survey: Part One

ANCHOR Survey: Part One A

We invite a representative from your organisation or service to take part in the Australian National Child Hearing Health Outcomes Registry (ANCHOR) Aim 1 Survey: *Mapping Australia's Hearing Health Services and Databases*.

What is ANCHOR?

ANCHOR is a 3-year project funded by the NHMRC and endorsed by the Department of Health and Age Care's Hearing Services Program.

ANCHOR's vision is to provide:

- A national child evidence base for future hearing policies, service delivery models and supports;
- A mechanism for improved models of service delivery and equity of access;
- A pathway to national reporting for educational outcomes; and
- A national platform to facilitate population-based research for DHH children

What are the aims of this survey?

The primary objective of this survey is to describe existing child hearing health services, databases and outcome measures across Australia.
The survey will contribute towards the overarching objective of the project by helping:

- Determine the feasibility of the proposed ANCHOR
- Plan data linkage between databases (Victorian and Queensland), and to inform the development of a model for a future national registry (other states).
- Determine a core set of outcomes to be measured for Australian deaf and hard of hearing children.

What will be the outcome of this survey?

The outcome of this survey will be a report that describes all existing Australian child hearing health-related services, databases and outcome measures, including:

- Data and database types,
- Current reporting arrangements,
- Coverage and quality of data,
- Ability to link to other data collections,
- Relevant policies, frameworks and procedures, and
- Stakeholder willingness to work towards ANCHOR.

Who is this survey for?

We invite a representative from every organisation offering hearing health, education, support, advocacy or research to deaf and hard of hearing children up to 18 years of age in Australia to complete the survey, including organisations or services that offer:

- Universal newborn hearing screening (UNHS)
- Post-UNHS hearing screening
- Diagnostic audiology
- Hearing rehabilitation service
- Early intervention specifically for DHH children
- Allied health (e.g. speech pathology, psychology, physiotherapy, occupational therapy, optometry, other)
- Medical services (e.g. paediatrics, ENT (Ear Nose Throat), genetics, ophthalmology either public or private)
- Education (preschool, primary or secondary)
- Parent support (either health professional support, parent mentoring, peer to peer support or other)
- Deaf advocacy
- Maternal and child health
- Aboriginal and Torres Strait Islander Health Service
- Research registry or longitudinal cohort

If you are not the key representative of your organisation, please discuss with your organisation lead who is the best person to fill in this survey.

Data storage and confidentiality

Due to the commercially sensitive nature of some questions, identifiable data from this report will not be shared. All your responses will be kept confidential, and no organisation will be identified in any publication of results. Only group level data will be reported. You and your organisation will have the opportunity to view the report of results prior to publication.

Data will be stored electronically on secure servers at the Murdoch Children's Research Institute in Melbourne.

Instructions

You can skip questions if you are not able to provide all types of information.

This survey will take up to one hour if your organisation provides one main hearing health service. If could take up to two hours if your organisation provides several hearing health services.

If you cannot complete the survey in one session your answers will be saved and you will receive a return code so you can return and complete the survey at a later time.

If you would like to know more about ANCHOR, please click for a pop up information box.

If you have any questions or prefer to complete the questionnaire in an online meeting format with researchers, please contact us by email anchor@mcri.edu.au or indicate on the following page.

Please continue to the survey.

Please tell us where your service/organisation operates (select all that apply):

☐ Australian Capital Territory
☐ New South Wales
☐ Northern Territory
☐ South Australia
☐ Tasmania
☐ Queensland
☐ Victoria
☐ Western Australia

This survey has two/three parts:

- **Part One:** About your organisation, services provided, information recorded and data governance. This section could be filled out by a key representative of your organisation. This could be a manager, health/education professional or senior administrator. We hope to use information from Part One to gain an understanding of existing child hearing datasets to help determine whether a child hearing national database may be possible in the future, what outcomes measures may be feasibly collected and identify barriers to its possible implementation.
- **Part Two:** About costs, including the estimated cost of staffing, data collection, data entry and database management and the potential cost of data systems change. This part comprises of a spreadsheet that we will send via email. Part two can be completed later by a staff member who has information about staff salaries and other costs. We hope to use information from Part Two to determine the feasibility and projected costs of adopting a future child hearing national database.
- **Part Three:** (VIC and QLD only) About the database and data governance. This section asks technical questions about your organisation's databases. This section can be completed by a data/database manager or IT officer, however if your organisation does not have a specific data person, please fill out the questions as best you can. We hope to use information from Part Three to plan data linkage between Victorian and Queensland data sets.

If you have any questions or prefer to complete the questionnaire in an online meeting format with researchers, please contact us by email anchor@mcri.edu.au or indicate below.

**Do you prefer to complete the survey in an online meeting and/or would you like someone from the ANCHOR team to contact you to answer questions about the survey?**	☐	Yes, I would like someone from the ANCHOR team to contact me
	☐	No, I will proceed to the survey now
**(If yes) When would you like us to contact you, and what is your preferred contact method (phone or email)? Please provide your contact details below.**		

Your contact details

**Your full name**	
**Name of your organisation/service**	
**Your role**	
**Email address**	
**Phone number**	

Consent

By ticking this box and continuing with the survey, I consent to:

1. **Participation in the Australian National Child Hearing health Outcomes Registry (ANCHOR) Aim 1 Survey.**
2. **Use of the information I provide:**
    a. **To help determine the feasibility of a National Child Hearing health Outcomes Registry**
    b. **To help determine a core set of outcomes to be measured for Australian deaf and hard of hearing children**
    c. **To plan data linkage between databases (Victorian and Queensland), OR to inform the development of a model for a future national registry (other states).**
3. **To be contacted by the ANCHOR team if they need to clarify any answers or need more information.**

☐ Yes ☐ No

I have received approval from the CEO/Director/Senior staff member at my organisation to participate in this survey on behalf of my organisation or service.

☐ Yes ☐ No

OR I do not require additional organisational approval to participate

☐ Yes

**Approving person's name**	
**Approving person's role**	
**Date approval given**	
**Research staff to upload email correspondence demonstrating organisation has approved participation in the Aim 1 survey.**	

Before we start

What is data?

This survey contains questions related to information or "data" that is collected and recorded by your organisation or service. To help you answer the questions as accurately as possible, the ANCHOR team defines data as ***any piece of information that is recorded about a client, whether it is personal, demographic, clinical, or service-related.*** For example:

- Personally identifying information (name, date of birth, Medicare number, phone number, email address, home address)
- Other demographic Information (e.g. Aboriginal and Torres Strait Islander status, language spoken)
- Health service use information (e.g. referrals and appointment attendance)

- Clinical information (e.g. audiology data, medical diagnoses, test results)
- Research information (e.g. information collected or used for research purposes)

Data can include information that is stored electronically in a database, or physically on paper records.

We will ask you whether your organisation/service routinely records various types of information in either of the following formats:

1. Data is recorded in a **field** (stored electronically)
2. Data is recorded in **notes only** (either electronically or on paper)

What is a field?

A field is an area of a database or electronic record which captures a single, specific piece of information or data.

Information can be entered into a field in a number of ways, including:

- Text (e.g. name)
- Numerical (e.g. a hearing threshold)
- From a date calendar (e.g. date of birth)
- From a dropbox menu (e.g. degree of hearing loss)
- Checkbox

A field differs from general notes is that you cannot or would not enter anything into a field besides the specific piece of information that the field is designed for. Additionally, notes can be either electronic or paper records, but fields are only in electronically stored data.

An example of data stored as notes is shown below.

"21/1/23 John's audiogram today indicates:

Right ear – severe sensorineural hearing loss

Left ear – normal hearing

The primary aetiology is enlarged vestibular aqueduct syndrome.

There is no family history of hearing loss."

Below you can see the same data contained in fields.

First name	John
Date of assessment	21/01/2023
Right ear hearing degree	Severe
Right ear hearing loss type	Sensorineural
Left ear hearing degree	Normal
Left ear hearing loss type	
Primary aetiology	Enlarged vestibular aqueduct syndrome
Family history of hearing loss	☐ YES ☒ NO

If you have any questions, please contact the ANCHOR team at anchor@mcri.edu.au.

The role of your organisation (please select all that apply):

- ☐ Universal newborn hearing screening (UNHS)
- ☐ Post-UNHS hearing screening
- ☐ Diagnostic audiology
- ☐ Hearing rehabilitation
- ☐ Hearing implantation
- ☐ Early intervention specifically for Deaf and hard of hearing children
- ☐ Allied health (e.g. speech pathology, psychology, physiotherapy, occupational therapy, optometry, other)
- ☐ Medical services (e.g. paediatrics, ENT (Ear, Nose and Throat), genetics, ophthalmology (public or private))
- ☐ Education provider (preschool, primary or secondary)
- ☐ Parent support (health professional support, parent mentoring, peer to peer support, or other)
- ☐ Deaf advocacy and support services
- ☐ Maternal and child health
- ☐ Aboriginal and Torres Strait Islander health or hearing service

☐ Research registry or longitudinal cohort
☐ Other (please specify)

If other, please specify

Population served (select all that apply)

	Yes	No
**Newborns/children only**		
**Newborn/children and adults**		
**Aboriginal and Torres Strait Islander children**		
**Parents or families of children with hearing loss (where the parent or family is the primary client)**		
**Only adults (18 years over) with hearing loss**		
**Other (please specify)**		

(If only adults were selected) You selected 'Adults with hearing loss only'. This survey aims to collect information from organisations/services relating to child hearing health only. Thank you for your time. Please select the box to exit the survey.

☐ **Exit the survey**

If other, please specify

Please specify age range of children that your organisation provides services to.

My organisation provides services for children with: (select all that apply)

☐ Sensorineural hearing loss
☐ Permanent conductive hearing loss
☐ Chronic conductive hearing loss
☐ Transient conductive hearing loss
☐ Auditory processing disorder
☐ All types of hearing loss

**How many individual sites does your organisation see children at?**	

Service/program setting (select all that apply)

	Yes	No
**Clinic – hospital setting**		
**Clinic – community setting (please specify)**		
**Hub (please specify)**		
**Remote/outreach**		
**Maternal and Child Health Nurse Centre**		
**Preschool/childcare**		
**School**		
**Home**		

**Internet/App based**		
**Phone**		
**Research centre**		
**Other (please specify)**		

If other, please specify

[ ]

Overview of the service/programs provided by your organisation to deaf and hard of hearing children and families.

If your organisation provides more than one service/program for deaf and hard of hearing children, or if the name of your service/program is different to the name of your organisation, please name each service/program here. You may add a web link here.

[ ]

Funding

What funding do you provide paediatric services under? (please select all that apply)

- [ ] Health
- [ ] Education
- [ ] National Disability Insurance Scheme (NDIS)
- [ ] Other social services
- [ ] Federal funding
- [ ] Private health insurance
- [ ] User pays
- [ ] Other

(If other) What other funding do you provide services under?

[ ]

Are you a registered NDIS provider?

- [ ] Yes
- [ ] No

About your service

We are interested to know the referral pathways in and out of your services/programs. This helps us better understand the flow of information between organisations and where we can access additional data about deaf and hard of hearing children.

For service-based programs: Where do you receive referrals from? If you have more than one service/program, please answer for all services/programs in your organisation.

For research programs: What services/settings do you recruit from?

Please respond "Yes" or "No" for each option.

	**Yes, accept**	**No, don't accept**

	**referrals from**	**referrals from**
Birthing hospitals		
Hearing screening sites/programs		
Diagnostic Audiologists		
Rehabilitation Audiologists		
Cochlear implant services		
Parent support programs (including professional, peer to peer or parent mentor programs)		
Teachers / Principals / Schools		
Teachers of the Deaf		
Early childhood educators/programs		
Early intervention programs for Deaf and Hard of Hearing Children		
Speech pathologists		
Occupational therapists		
Psychologists		
Physiotherapists		
Social workers		
Other early intervention/allied health		
Nurses/Midwives/Child Health Nurses/MCHNs		
Indigenous Health Workers		
General practitioners		
Paediatricians		
Geneticists		
Ear Nose and Throat Specialists		
Ophthalmologists/Optometrists		
Other medical specialists		
Parents/carers		
Researchers		
The community/no referral criteria		
Other (please specify)		

If other, please specify ______

For service-based programs: Where do you send client information to? If you have more than one service/program, please answer for all services/programs in your organisation.

Please respond "Yes" or "No" for each option.

	**Yes, accept referrals from**	**No, don't accept referrals from**
Hearing screening sites/programs		
Diagnostic Audiologists		

Rehabilitation Audiologists		
Cochlear implant services		
Parent support programs (including professional, peer to peer or parent mentor programs)		
Teachers / Principals / Schools		
Teachers of the Deaf		
Early childhood educators/programs		
Early intervention programs for Deaf and Hard of Hearing Children		
Speech pathologists		
Occupational therapists		
Psychologists		
Physiotherapists		
Social workers		
Other early intervention/allied health		
Nurses/Midwives/Child Health Nurses/MCHNs		
Indigenous Health Workers		
General practitioners		
Paediatricians		
Geneticists		
Ear Nose and Throat Specialists		
Ophthalmologists/Optometrists		
Other medical specialists		
Parents/carers		
Researchers		
The community/no referral criteria		
Other (please specify)		

If other, please specify

About your service

We are interested in who collects and enters data within your organisation, so we may be able to estimate the cost of any potential changes to workflows.

Who COLLECTS data about deaf and hard of hearing children?

Please respond “Yes” or “No” for each option.

	Yes, accept referrals from	No, don’t accept referrals from
Diagnostic Audiologists		
Rehabilitation Audiologists		
Cochlear implant services		

Parent support programs (including professional, peer to peer or parent mentor programs)		
Teachers / Principals / Schools		
Teachers of the Deaf		
Early childhood educators/programs		
Early intervention programs for Deaf and Hard of Hearing Children		
Speech pathologists		
Occupational therapists		
Psychologists		
Physiotherapists		
Social workers		
Other early intervention/allied health		
Nurses/Midwives/Child Health Nurses/MCHNs		
Indigenous Health Workers		
General practitioners		
Paediatricians		
Geneticists		
Ear Nose and Throat Specialists		
Ophthalmologists/Optometrists		
Other medical specialists		
Parents/carers		
Researchers		
The community/no referral criteria		
Other (please specify)		
**If other, please specify**		

Who ENTERS data into databases about deaf and hard of hearing children?

Please respond "Yes" or "No" for each option.

	**Yes, accept referrals from**	**No, don't accept referrals from**
Diagnostic Audiologists		
Rehabilitation Audiologists		
Cochlear implant services		
Parent support programs (including professional, peer to peer or parent mentor programs)		
Teachers / Principals / Schools		
Teachers of the Deaf		
Early childhood educators/programs		
Early intervention programs for Deaf and Hard of Hearing Children		

Speech pathologists		
Occupational therapists		
Psychologists		
Physiotherapists		
Social workers		
Other early intervention/allied health		
Nurses/Midwives/Child Health Nurses/MCHNs		
Indigenous Health Workers		
General practitioners		
Paediatricians		
Geneticists		
Ear Nose and Throat Specialists		
Ophthalmologists/Optometrists		
Other medical specialists		
Parents/carers		
Researchers		
The community/no referral criteria		
Other (please specify)		

If other, please specify ☐

Data reporting

We are interested in understanding the kind of reporting your organisation undertakes and whether data in these reports could be used for future data linkage activities.

Does your organisation produce any routine reports/summaries using data collected from clients? (For example, monthly/quarterly summary statistics, number of referrals received, number of referrals received, number of clients seen)

☐ Yes ☐ No

If yes, please provide information on the frequency and type of aggregate reporting (for example, reports containing summary of numbers of children seen in the service or number of children with particular diagnoses or characteristics

☐

**Please upload an example of an aggregate report if it can be shared**	

Please provide information on the frequency and type of individual (child) level reporting (For example, a report containing individual screening or test results for all children seen that month, or number of appointments attended/missed for each client)

☐

**Please upload an example of an individual de-identified report if it can be shared**	

Are these reports provided to any external funding bodies or other organisations?

☐ Yes ☐ No

If yes, please provide details. ☐

Data Storage

How does your organisation routinely records* the following information, by selecting one of the of the following:

- **Routinely recorded in an electronic field**
- **Routine recorded in notes only (either electronically or on paper)**
- **Not routinely recorded**

***'routinely record' means that your organisation usually records this information for every child, if it is available.**

	Routinely recorded in an electronic field	Routinely recorded in notes only (paper or electronic)	Not routinely recorded
**Personally identifying information (e.g. name, DOB, Medicare number, contact details)**			
**Other demographic Information (e.g. sex, Aboriginal and Torres Strait Islander status, language spoken)**			
**Health service use information (e.g. appointment dates, failure to attend)**			
**Clinical information (e.g. audiology results, medical diagnoses, test results)**			
**Research information (e.g. information collected for research purposes)**			

If any data is stored electronically, we would like to know more about <u>your primary database</u>. Before we ask about your primary database, does your organisation provide or enter data into any databases maintained by other teams or organisations?

☐ Yes ☐ No

If yes, please provide the name of any other database(s) that you provide data for, and who manages the database(s). We will contact them directly for information. For the rest of this survey, we are interested in <u>your teams or organisation's database(s).</u>

☐

**Name of your organisation's primary database**	
**Purpose of database**	

Do you have a data dictionary for this database?
(A data dictionary is a collection of names, definitions, and attributes about data elements that are being used or captured in a database. It describes the meanings and purposes of data elements and provides guidance on interpretation, accepted meanings and representation.)

☐ Yes ☐ No

If yes, can you provide us with a copy of your data dictionary?

If yes (to the above), what format is it in, and is it readily accessible to data users?

Do you have a schema/list of data fields collected?

☐ Yes ☐ No

Do you have more than one database?

☐ Yes ☐ No

(If yes)

**Name of your organisation's secondary database**	
**Purpose of this database**	

Do you have a data dictionary for this database?
(A data dictionary is a collection of names, definitions, and attributes about data elements that are being used or captured in a database. It describes the meanings and purposes of data elements and provides guidance on interpretation, accepted meanings and representation.)

☐ Yes ☐ No

If yes, can you provide us with a copy of your data dictionary?

If yes (to the above), what format is it in, and is it readily accessible to data users?

Do you have a schema/list of data fields collected?

☐ Yes ☐ No

Is there a minimum dataset (data that is collected for every client) that is maintained?

☐ Yes ☐ No

(If yes) Please describe what is included, or indicate in your data dictionary/schema.

What time period does the minimum dataset cover (for example, from 2016 onwards)?

Please email a copy of your data dictionary and schema to: anchor@mcri.edu.au.
If you are unable to share it with us via e-mail, please let us know and we will get in contact with you.
Please note that we may contact you for further information once we have reviewed your data dictionary.

ANCHOR Survey Part One B

We would like to explore whether future data linkage with your organisation is possible. We would like to understand the types of information you routinely record about deaf and hard of hearing children.
Including:

- **Personally identifying information (this refers to name, date of birth, Medicare number, phone number, email address and home address)**
- **Other demographic Information (e.g. sex, Aboriginal and Torres Strait Islander status, language spoken)**
- **Health service use information (e.g. referrals, appointment attendance)**
- **Clinical information (e.g. audiology data, medical diagnoses, test results)**
- **Research information (e.g. information collected or used for research purposes)**

This helps us understand what possible types of data could be minimally collected in the future for all hearing health services.

What personally identifying information (PII) is collected from clients of your service/program in your primary database N/A?
Please indicate if you routinely record* the following information, by selecting one of the following:

- **Routinely recorded in an electronic field**
- **Routinely recorded in notes only (either electronically or on paper)**
- **Not routinely recorded**

***'routinely record' means that your organisation usually records this information for every child, if it is available.**

	Routinely recorded in an electronic field	Routinely recorded in notes only (paper or electronic)	Not routinely recorded
**Name (first and last name)**			
**Date of birth**			
**Medicare number**			

**Primary parent/caregiver phone number**			
**Primary parent/caregiver email address**			
**Postcode**			

Demographic Information

Please indicate if you routinely record* the following information, by selecting one of the following:

- **Routinely recorded in an electronic field**
- **Routinely recorded in notes only (either electronically or on paper)**
- **Not routinely recorded**

***'routinely record' means that your organisation usually records this information for every child, if it is available.**

	Routinely recorded in an electronic field	Routinely recorded in notes only (paper or electronic)	Not routinely recorded
**Sex/gender**			
**Country of birth**			
**State or territory of birth**			
**Aboriginal and/or Torres Strait Islander status**			
**Deceased**			
**Date deceased**			

Demographic Information

Please indicate if you routinely record* the following information, by selecting one of the following:

- **Routinely recorded in an electronic field**
- **Routinely recorded in notes only (either electronically or on paper)**
- **Not routinely recorded**

***'routinely record' means that your organisation usually records this information for every child, if it is available.**

	Routinely recorded in an electronic field	Routinely recorded in notes only (paper or electronic)	Not routinely recorded
**Date of arrival in Australia (if country of birth is not Australia)**			
**Refugee status**			
**Citizenship/visa status**			
**Out of home care**			
**Child protection involvement**			
**Medical alerts**			

Demographic Information

Please indicate if you routinely record* the following information, by selecting one of the following:

- **Routinely recorded in an electronic field**
- **Routinely recorded in notes only (either electronically or on paper)**
- **Not routinely recorded**

***'routinely record' means that your organisation usually records this information for every child, if it is available.**

	Routinely recorded in an electronic field	Routinely recorded in notes only (paper or electronic)	Not routinely recorded
**Primary language spoken at home**			
**Secondary/other language spoken to home**			
**Primary parent/caregiver language spoken**			
**Secondary parent/caregiver language spoken**			
**Child/client language spoken**			
**Communication mode**			

Demographic Information

Please indicate if you routinely record* the following information, by selecting one of the following:

- **Routinely recorded in an electronic field**
- **Routinely recorded in notes only (either electronically or on paper)**
- **Not routinely recorded**

***'routinely record' means that your organisation usually records this information for every child, if it is available.**

	Routinely recorded in an electronic field	Routinely recorded in notes only (paper or electronic)	Not routinely recorded
**Primary parent/caregiver first and last name**			
**Primary parent/caregiver address**			
**Primary parent/caregiver relationship to child**			
**Secondary parent/caregiver first and last name**			
**Secondary parent/caregiver address**			
**Secondary parent/caregiver relationship to child**			
**Household composition/participant/client/student living arrangements (single parent, partnered, number of children)**			
**Household income**			

**Parent/caregiver Health Care Card holder**			
**Primary parent/caregiver country of birth**			
**Second parent/caregiver country of birth**			
**Primary parent/caregiver education level**			
**Secondary parent/caregiver education level**			
**Primary parent/caregiver occupation or parent occupation group**			
**Secondary parent/caregiver occupation or parent occupation group**			

Demographic Information

Please indicate if you routinely record* the following information, by selecting one of the following:

- **Routinely recorded in an electronic field**
- **Routinely recorded in notes only (either electronically or on paper)**
- **Not routinely recorded**

***'routinely record' means that your organisation usually records this information for every child, if it is available.**

	Routinely recorded in an electronic field	Routinely recorded in notes only (paper or electronic)	Not routinely recorded
**Gestational age at birth**			
**Birth weight**			
**Single/twin/multiple birth**			
**Order of child in family**			

Service provision Information

Please indicate if you routinely record* the following information, by selecting one of the following:

- **Routinely recorded in an electronic field**
- **Routinely recorded in notes only (either electronically or on paper)**
- **Not routinely recorded**

***'routinely record' means that your organisation usually records this information for every child, if it is available.**

	Routinely recorded in an electronic field	Routinely recorded in notes only (paper or electronic)	Not routinely recorded
**Medicare status (eligible/not eligible)**			
**NDIS status**			

**Funding source (e.g. private/public/health insurance/specific funding program)**			
**Number of encounters/occasions of service**			
**Hours of service provided**			

Does your organisation collect information on hearing loss risk factors?

☐ Yes ☐ No

(If yes), please indicate if you routinely record* the following information about risk factors by selecting one of the following:

- **Routinely recorded in an electronic field**
- **Routinely recorded in notes only (either electronically or on paper)**
- **Not routinely recorded**

***'routinely record' means that your organisation usually records this information for every child, if it is available.**

	Routinely recorded in an electronic field	Routinely recorded in notes only (paper or electronic)	Not routinely recorded
**Risk factor: family history of hearing loss**			
**Risk factor: syndrome**			
**Risk factor: ventilation/prolonged ventilation**			
**Risk factor: NICU admission**			
**Risk factor: low birthweight**			
**Risk factor: bacterial meningitis**			
**Risk factor: encephalitis**			
**Risk factor: asphyxia**			
**Risk factor: craniofacial abnormalities**			
**Risk factor: hyperbilirubinaemia**			
**Risk factor: TORCH infection/ perinatal infection**			
**Risk factor: significant head injury**			
**Risk factor: neurodegenerative disorder**			
**Risk factor: ototoxic medications**			
**Risk factor: professional concern**			
**Risk factor: other (please specify)**			

If other, please specify

You indicated that your organisation provides the following service(s):

We will now ask some questions about the specific service-related information that your organisation records.

Questions for newborn hearing screening programs

Are there any differences in the data you collect for "Target condition" versus "Non-target condition" newborns?

☐

What screening device does your program use?

☐

What aABR stimulus does your screening program use?

☐ Click ☐ Broadband chirp

What screening pass level (dBnHL) does your program use (i.e. the stimulus level used in the aABR screen)?

☐

Please indicate if you routinely record* the following information by selecting one of the following:

- **Routinely recorded in an electronic field**
- **Routinely recorded in notes only (either electronically or on paper)**
- **Not routinely recorded**

***'routinely record' means that your organisation usually records this information for every child, if it is available.**

	Routinely recorded in an electronic field	Routinely recorded in notes only (paper or electronic)	Not routinely recorded
**Birth hospital/facility**			
**Screening hospital/location/facility**			
**Age at screen**			
**Corrected age**			
**Screen date(s)**			
**Right ear result**			
**Left ear result**			

Do you collect downstream data, such as referrals or diagnostic audiology results?

☐ Yes ☐ No

(If yes) Data collected from other services by screening services

Please indicate if you routinely record* the following information, by selecting one of the following:

- **Routinely recorded in an electronic field**
- **Routinely recorded in notes only (either electronically or on paper)**
- **Not routinely recorded**

***'routinely record' means that your organisation usually records this information for every child, if it is available.**

	Routinely recorded in an electronic field	Routinely recorded in notes only (paper or electronic)	Not routinely recorded
**Date of diagnostic audiology referral**			
**Name of diagnostic audiology service**			
**Audiology status**			
**Date of initial diagnosis**			
**Date of early intervention (EI) referral**			
**Date of EI enrolment**			
**Name of EI agency**			
**Date of Hearing Australia referral**			
**Date of first Hearing Australia appointment**			
**Date of hearing device fitting**			
**Hearing Australia branch**			
**Date of CI referral**			
**Name of CI service**			
**CI initial appointment date**			
**Implant 1 date**			
**Implant 2 date**			

Audiology Diagnostic Summary (for right ear and left ear)

Please indicate whether your organisation routinely records* the following information by selecting one of the following:

- **Routinely recorded in an electronic field**
- **Routinely recorded in notes only (either electronically or on paper)**
- **Not routinely recorded**

***'routinely record' means that your organisation usually records this information for every child, if it is available.**

	Routinely recorded in an electronic field	Routinely recorded in notes only (paper or electronic)	Not routinely recorded
**Permanent, temporary, unknown**			
**Type of hearing loss (SNHL, ANSD, permanent conductive, mixed)**			
**Atresia/Microtia (yes/no)**			

**Degree of hearing loss (e.g. mild, moderate, severe, profound)**			
**3 frequency average hearing loss**			
**4 frequency average hearing loss**			

Please tell us how your organisation categorises degrees of hearing loss and the lower and upper limits for each category.
Please indicate the categories that your organisation uses (select all that apply).

- ☐ Normal
- ☐ Slight
- ☐ Mild
- ☐ Moderate
- ☐ Moderately severe
- ☐ Severe
- ☐ Severe to profound
- ☐ Profound
- ☐ Other

**Upper limit of the Normal range**	
**Lower limit of the Slight range**	
**Upper limit of the Slight range**	
**Lower limit of the Mild range**	
**Upper limit of the Mild range**	
**Lower limit of the Moderate range**	
**Upper limit of the Moderate range**	
**Lower limit of the Moderately Severe range**	
**Upper limit of the Moderately Severe range**	
**Lower limit of the Severe range**	
**Upper limit of the Severe range**	
**Lower limit of the Severe to Profound range**	
**Upper limit of the Severe to Profound range**	
**Lower limit of the Profound range**	

Below we ask whether your organisation records detailed audiology information.
We understand that not all information will be recorded for every child because the tests that are conducted depend on both the clinical presentation of the child, and also the testing situation.
We would like to know what information you can record in your database/paper records.

Do you record frequency-specific electrophysiological test results?

- ☐ Yes
- ☐ No

(If yes)

Do you record ASSR thresholds?

- ☐ Yes
- ☐ No

(If yes) please indicate how you routinely record* the following information by selecting one of the following:

- **Routinely recorded in an electronic field**
- **Routinely recorded in notes only (either electronically or on paper)**
- **Not routinely recorded**

***'routinely record' means that your organisation usually records this information for every child, if it is available.**

	Routinely recorded in an electronic field	Routinely recorded in notes only (paper or electronic)	Not routinely recorded
**Air conduction thresholds (dBnHL, unmasked)**			
**Air conduction thresholds (dBeHL, unmasked)**			
**Air conduction thresholds (dBnHL, masked)**			
**Air conduction thresholds (dBeHL, masked)**			
**Bone conduction thresholds (dBnHL, unmasked)**			
**Bone conduction thresholds (dBeHL, unmasked)**			
**Bone conduction thresholds (dBnHL,nmasked)**			
**Bone conduction thresholds (dBeHL, masked)**			
**Diagnostic equipment brand/model**			
**Stimulus (e.g. 90Hz)**			
**Transducer (e.g. headphone, insert)**			
**Other (please specify)**			

(if other) what other ASSR information do you record?

Please comment on whether you attempt to collect all four thresholds (0.5, 1, 2 & 4kHz) on ASSR testing if possible/ where indicated.

(If yes)

Do you record frequency specific ABR thresholds? ☐ Yes ☐ No

(If yes) please indicate how you routinely record* the following information by selecting one of the following:

- **Routinely recorded in an electronic field**
- **Routinely recorded in notes only (either electronically or on paper)**

- **Not routinely recorded**

***'routinely record' means that your organisation usually records this information for every child, if it is available.**

	Routinely recorded in an electronic field	Routinely recorded in notes only (paper or electronic)	Not routinely recorded
**Air conduction thresholds (dBnHL, unmasked)**			
**Air conduction thresholds (dBeHL, unmasked)**			
**Air conduction thresholds (dBnHL, masked)**			
**Air conduction thresholds (dBeHL, masked)**			
**Bone conduction thresholds (dBnHL, unmasked)**			
**Bone conduction thresholds (dBeHL, unmasked)**			
**Bone conduction thresholds (dBnHL,nmasked)**			
**Bone conduction thresholds (dBeHL, masked)**			
**Diagnostic equipment brand/model**			
**Stimulus (e.g. toneburst, chirp)**			
**Transducer (e.g. headphone, insert)**			
**Other (please specify)**			

(if other) what other frequency specific ABR information do you record?

Please comment on whether you attempt to collect all four thresholds (0.5, 1, 2 & 4kHz) on frequency specific ABR testing if possible/ where indicated.

Do you record results of ABR testing using a broadband stimulus?

☐ Yes
☐ No

(if yes) Please indicate how you routinely record* the following information by selecting one of the following:

- **Routinely recorded in an electronic field**
- **Routinely recorded in notes only (either electronically or on paper)**
- **Not routinely recorded**

***'routinely record' means that your organisation usually records this information for every child, if it is available.**

	Routinely recorded in an electronic field	Routinely recorded in notes only (paper or electronic)	Not routinely recorded
**Air conduction threshold (dBnHL)**			
**Air conduction threshold (dBeHL)**			
**Bone conduction threshold (dBnHL)**			
**Bone conduction threshold (dBeHL)**			
**Bone conduction thresholds (dBnHL,nmasked)**			
**Supra-threshold test levels**			
**Wave latencies**			
**Masked/unmasked**			
**Diagnostic equipment brand/model**			
**Stimulus (e.g. click, chirp)**			
**Transducer (e.g. headphone, insert)**			
**Other (please specify)**			

(if other), what other broadband ABR information do you record?

Do you record behavioural audiometry thresholds?

☐ Yes
☐ No

(if yes) Please indicate how you routinely record* the following information by selecting one of the following:

- **Routinely recorded in an electronic field**
- **Routinely recorded in notes only (either electronically or on paper)**
- **Not routinely recorded**

***'routinely record' means that your organisation usually records this information for every child, if it is available.**

	Routinely recorded in an electronic field	Routinely recorded in notes only (paper or electronic)	Not routinely recorded
**Air conduction threshold (left/right) unmasked**			
**Air conduction threshold (left/right) masked**			
**Air conduction threshold (freefield)**			

**Bone conduction threshold (left/right) unmasked**			
**Bone conduction thresholds (left/right) masked**			
**Bone conduction threshold (ear not specified)**			
**Continuous audiogram**			
**Behavioural observation (BOA) results**			
**Method (e.g. VROA, play)**			
**Diagnostic equipment brand/model (e.g. audiometer)**			
**Stimulus (e.g. pure tone, warble tone)**			
**Transducer (e.g. headphone, insert)**			
**Other (please specify)**			

(if other), what other behavioural audiometry information do you record?

Please indicate what other audiology tests results you record:

- ☐ **TEOAE**
- ☐ **DPOAE**
- ☐ **226Hz tympanometry**
- ☐ **Other tympanometry (e.g. 1000Hz, multifrequency)**
- ☐ **Cochlear microphonics**
- ☐ **Speech audiometry**
- ☐ **Other**
- ☐ **None**

(if yes to TEOAE) Please indicate how you routinely record* the following information about TEOAEs, by selecting one of the following:

- **Routinely recorded in an electronic field**
- **Routinely recorded in notes only (either electronically or on paper)**
- **Not routinely recorded**

***'routinely record' means that your organisation usually records this information for every child, if it is available.**

	Routinely recorded in an electronic field	Routinely recorded in notes only (paper or electronic)	Not routinely recorded

**Response frequency range**			
**Signal to noise ratio**			
**Response reproducibility**			
**Overall result (present, absent, unobtainable)**			
**Diagnostic equipment brand/model**			
**Other (please specify)**			

(if other) What other TEOAE information do you record?

(if yes to DPOAE) Please indicate how you routinely record* the following information about DPOAEs, by selecting one of the following:

- **Routinely recorded in an electronic field**
- **Routinely recorded in notes only (either electronically or on paper)**
- **Not routinely recorded**

***'routinely record' means that your organisation usually records this information for every child, if it is available.**

	Routinely recorded in an electronic field	Routinely recorded in notes only (paper or electronic)	Not routinely recorded
**Present/absent/unobtainable**			
**Frequency range**			
**DP level**			
**Noise level**			
**Signal to noise ratio**			
**Diagnostic equipment brand/model**			
**Other (please specify)**			

(if other) What other DPOAE information do you record?

(if yes to 226Hz tympanometry) Please indicate how you routinely record* the following information about 226Hz tympanometry, by selecting one of the following:

- **Routinely recorded in an electronic field**
- **Routinely recorded in notes only (either electronically or on paper)**
- **Not routinely recorded**

***'routinely record' means that your organisation usually records this information for every child, if it is available.**

	Routinely recorded in an electronic field	Routinely recorded in notes only (paper or electronic)	Not routinely recorded
**Type (e.g. A, B, C)**			
**Subtype (e.g. As, Ad, B(low), B(high), C1, C2)**			
**Middle ear pressure**			
**Static compliance**			
**Ear canal volume**			
**Tympanometer brand/model**			
**Other (please specify)**			

(if other) What other 226Hz tympanometry information do you record?

(if yes to other tympanometry - 1000Hz, multifrequency) Please indicate how you routinely record* the following information about other tympanometry (e.g. 1000Hz, multifrequency) by selecting one of the following:

- **Routinely recorded in an electronic field**
- **Routinely recorded in notes only (either electronically or on paper)**
- **Not routinely recorded**

***'routinely record' means that your organisation usually records this information for every child, if it is available.**

	Routinely recorded in an electronic field	Routinely recorded in notes only (paper or electronic)	Not routinely recorded
**Probe tone frequency**			
**Peak/no peak**			
**Indeterminate/unobtainable**			
**Tympanometer brand/model**			
**Other (please specify)**			

(if other) What other information do you record for 'other' tympanometry?

Please select what probe tone frequencies you use (select all that apply)

- ☐ 1000Hz
- ☐ 678Hz
- ☐ Multifrequency

(if yes to cochlear microphonics) Please indicate how you routinely record* the following information about other tympanometry (e.g. 1000Hz, multifrequency) by selecting one of the following:

- **Routinely recorded in an electronic field**
- **Routinely recorded in notes only (either electronically or on paper)**
- **Not routinely recorded**

***'routinely record' means that your organisation usually records this information for every child, if it is available.**

	Routinely recorded in an electronic field	Routinely recorded in notes only (paper or electronic)	Not routinely recorded
**Stimulus (e.g. click)**			
**Stimulus level**			
**Present/absent/unobtainable**			
**Diagnostic equipment brand/model**			
**Other (please specify)**			

(if other) What other cochlear microphonic information do you record?

(if yes to speech audiometry) Please indicate how you routinely record* the following information about speech audiometry, by selecting one of the following:

- **Routinely recorded in an electronic field**
- **Routinely recorded in notes only (either electronically or on paper)**
- **Not routinely recorded**

***'routinely record' means that your organisation usually records this information for every child, if it is available.**

	Routinely recorded in an electronic field	Routinely recorded in notes only (paper or electronic)	Not routinely recorded
**Type of speech test (e.g. AB word list)**			
**Live voice/recorded speech**			
**Speech presentation level**			
**Presented in quiet or in noise**			
**Noise level/signal to noise ratio**			
**Score**			
**Other (please specify)**			

(if other) What other speech audiometry information do you record?

(if yes to other) Please provide details of any other diagnostic audiology tests that you collect information on.

Do you have any comments about these other tests?

Questions for postnatal hearing screening organisations/services

What hearing conditions does your service screen for? (please select all that apply)

- ☐ Permanent hearing loss
- ☐ Temporary conductive hearing loss
- ☐ Middle ear dysfunction

Please indicate whether your organisation routinely records* the following information by selecting one of the following:

- **Routinely recorded in an electronic field**
- **Routinely recorded in notes only (either electronically or on paper)**
- **Not routinely recorded**

***'routinely record' means that your organisation usually records this information for every child, if it is available.**

	Routinely recorded in an electronic field	Routinely recorded in notes only (paper or electronic)	Not routinely recorded
**Referral date**			
**Referral reason**			
**Screening location/facility**			
**Age at screen**			
**Screen date(s)**			
**Right ear overall result (pass/refer)**			
**Left ear result overall result (pass/refer)**			
**Onward referral (e.g. to audiology, GP)**			

Please indicate if you use any of the following questionnaires, checklists or applications to screen hearing, speech, language, cognition, or other aspects of development.

- ☐ PEACH
- ☐ TEACH
- ☐ HATS
- ☐ PLUM
- ☐ FLI-P

☐ LittlEARS
☐ LIFE
☐ Other
☐ None

(If other), which other questionnaires, checklists or application does your service use?

Below we ask whether your organisation records detailed screening outcomes.
We understand that not all information will be recorded for every child because the screens that are conducted depend on both the presentation of the child, and also the testing situation.
We would like to know what information you can record in your database/paper records when following your organisation's protocols.

Do you record pure tone screen results?

☐ Yes
☐ No

If yes, please indicate how you routinely record* the following information by selecting one of the following:

- **Routinely recorded in an electronic field**
- **Routinely recorded in notes only (either electronically or on paper)**
- **Not routinely recorded**

***'routinely record' means that your organisation usually records this information for every child, if it is available.**

	Routinely recorded in an electronic field	Routinely recorded in notes only (paper or electronic)	Not routinely recorded
**Right/left results (pass/refer/unable to screen)**			
**Individual frequency result (pass/refer/unable to screen)**			
**Individual frequency threshold (dBHL)**			
**Equipment (e.g. audiometer brand, model)**			
**Stimulus (e.g. warble tone, pure tone)**			
**Transducer (e.g. headphone, insert)**			
**Other pure tone screen details**			

Please specify frequencies routinely screened and the pass level for each frequency.

Do you record results from other behavioural hearing screening methods (e.g. app-based screens)?

☐ Yes
☐ No

If yes, please indicate how you routinely record* the following information by selecting one of the following:

- **Routinely recorded in an electronic field**
- **Routinely recorded in notes only (either electronically or on paper)**
- **Not routinely recorded**

***'routinely record' means that your organisation usually records this information for every child, if it is available.**

	Routinely recorded in an electronic field	Routinely recorded in notes only (paper or electronic)	Not routinely recorded
**Overall result (pass/refer/unable to screen)**			
**Right/left result (pass/refer/unable to screen)**			
**Other screen details**			

(if other) please indicate what other behavioural screen details your organisation records, and how:

Do you record otoacoustic emission (OAE) screen results? ☐ Yes ☐ No

If yes, please indicate how you routinely record* the following information by selecting one of the following:

- **Routinely recorded in an electronic field**
- **Routinely recorded in notes only (either electronically or on paper)**
- **Not routinely recorded**

***'routinely record' means that your organisation usually records this information for every child, if it is available.**

	Routinely recorded in an electronic field	Routinely recorded in notes only (paper or electronic)	Not routinely recorded
**Transient evoked otoacoustic emission (TEOAE) screen result (pass/refer/unable to screen)**			
**Distortion product otoacoustic emission (DPOAE) screen result (pass/refer/unable to screen)**			
**Equipment (e.g. OAE screener brand/model)**			
**Other**			

(If other) please indicate what other OAE screen information your organisation records, and how:

Do you record tympanometry screen results?

☐ Yes
☐ No

If yes, please indicate how you routinely record* the following information by selecting one of the following:

- **Routinely recorded in an electronic field**
- **Routinely recorded in notes only (either electronically or on paper)**
- **Not routinely recorded**

***'routinely record' means that your organisation usually records this information for every child, if it is available.**

	Routinely recorded in an electronic field	Routinely recorded in notes only (paper or electronic)	Not routinely recorded
**Right/left ear overall result (pass/refer/unable to screen)**			
**Type (e.g. A, B, C)**			
**Subtype (e.g. As, Ad, B(low), B(high), C1, C2)**			
**Equipment (e.g. tympanometer brand/model)**			
**Other**			

(If other) please indicate what other tympanometry screen information your organisation records, and how:

Does your organisation collect downstream data, such as onward referrals or diagnostic audiology results?

☐ Yes
☐ No

If yes, please indicate how you routinely record* the following information by selecting one of the following:

- **Routinely recorded in an electronic field**
- **Routinely recorded in notes only (either electronically or on paper)**
- **Not routinely recorded**

***'routinely record' means that your organisation usually records this information for every child, if it is available.**

	Routinely recorded in an electronic field	Routinely recorded in notes only (paper or electronic)	Not routinely recorded
**Date of diagnostic audiology referral**			
**Name of diagnostic audiology service**			
**Audiology status**			

**Date of initial diagnosis**			
**Date of final diagnosis**			
**Date of referral to early intervention service**			
**Name of early intervention agency**			
**Date of early intervention enrolment**			
**Date of Hearing Australia referral**			
**Date of first Hearing Australia appointment**			
**Date of hearing device fitting**			
**Hearing Australia branch**			
**Date of hearing implant service referral**			
**Name of hearing implant service**			
**Hearing implant initial appointment date**			
**Implant 1 date**			
**Implant 2 date**			

Audiology Diagnostic Summary (for right ear and left ear)
Please indicate if you routinely record* the following information by selecting one of the following:

- **Routinely recorded in an electronic field**
- **Routinely recorded in notes only (either electronically or on paper)**
- **Not routinely recorded**

***'routinely record' means that your organisation usually records this information for every child, if it is available.**

	Routinely recorded in an electronic field	Routinely recorded in notes only (paper or electronic)	Not routinely recorded
**Type of hearing loss (SNHL, ANSD, permanent conductive, mixed, transient conductive)**			
**Degree of hearing loss (e.g. mild, moderate, severe, profound)**			
**Permanent, temporary, unknown**			

**3 or 4 Frequency Average Hearing Loss**			

Questions for hearing diagnostic services

Please indicate whether your organisation routinely records* the following information by selecting one of the following:

- **Routinely recorded in an electronic field**
- **Routinely recorded in notes only (either electronically or on paper)**
- **Not routinely recorded**

***'routinely record' means that your organisation usually records this information for every child, if it is available.**

	Routinely recorded in an electronic field	Routinely recorded in notes only (paper or electronic)	Not routinely recorded
**Client status (active/lost etc.)**			
**Referral date**			
**Referral reason**			
**Site/centre attended**			
**Age/date at first appointment**			
**Age/date of hearing loss confirmation/diagnosis**			
**Assessment discharge rate**			
**Failure to attend appointment**			
**Aetiology (primary)**			
**Aetiology (secondary)**			
**Progressive or fluctuating loss**			
**Co-morbidities**			

Please indicate if you use any of the following questionnaires, checklists or applications to screen hearing, speech, language, cognition, or other aspects of development.

- ☐ PEACH
- ☐ TEACH
- ☐ HATS
- ☐ PLUM
- ☐ FLI-P
- ☐ LittlEARS
- ☐ LIFE
- ☐ Other
- ☐ None

(If other) Which other questionnaires, checklists or applications does your service use?

Questions for hearing rehabilitation services

Please indicate how you routinely record* the following information, by selecting one of the following:

- **Routinely recorded in an electronic field**
- **Routinely recorded in notes only (either electronically or on paper)**
- **Not routinely recorded**

***'routinely record' means that your organisation usually records this information for every child, if it is available.**

Information about the child

	Routinely recorded in an electronic field	Routinely recorded in notes only (paper or electronic)	Not routinely recorded
**Client status (active/lost etc)**			
**Referral date**			
**Referral reason**			
**Site/centre attended**			
**Age/date at first appointment**			
**Age/date of hearing loss confirmation/diagnosis**			
**Failure to attend appointment**			
**Aetiology (primary)**			
**Aetiology (secondary)**			
**Progressive or fluctuating loss**			
**Comorbidities**			
**Communication approach (e.g. Auslan, spoken language)**			
**Parent participation or engagement**			
**COSI/goal setting**			

Amplification information

	Routinely recorded in an electronic field	Routinely recorded in notes only (paper or electronic)	Not routinely recorded
**Age/date of initial fitting (right ear/left ear)**			

**Current amplification device(s) (e.g. type, model)**			
**Previous amplification device(s)**			
**Hearing device serial number(s)**			
**Programmed hearing aid parameters**			
**Hearing device compliance/use/datalogging**			
**Real ear measurements**			
**Coupler gain**			
**Aided audiogram**			

Hearing implant information (e.g. cochlear implant, bone anchored hearing aid)

	Routinely recorded in an electronic field	Routinely recorded in notes only (paper or electronic)	Not routinely recorded
**Pre-implantation audiology results**			
**Date hearing implant evaluation**			
**Hearing implant candidacy (eligible/ineligible)**			
**Hearing implant recommendation (e.g. type of implant/unilateral/bilateral)**			
**Family decision (e.g. proceed/don't proceed)**			
**Reason for not proceeding with implant**			
**Hearing implant surgery date(s)**			
**Hearing implant switch on date(s)**			
**Implanted device (e.g. electrode array, abutment)**			
**Hearing implant processor**			
**Hearing implant use/compliance**			
**Mode (e.g. unilateral/bilateral/bimodal)**			

**Aided audiogram (with implant)**			

Other hearing device information

	Routinely recorded in an electronic field	Routinely recorded in notes only (paper or electronic)	Not routinely recorded
**Remote microphone system fitted**			
**Remote microphone system use**			
**Other assistive listening device(s) fitted**			
**Other accessory or assistive listening device usage/details (e.g. app)**			

Below we ask whether this hearing rehabilitation service records detailed audiology information.

We understand that not all information will be recorded for every child because the tests that are conducted depend on both the clinical presentation of the child, and also the testing situation.

We would like to know what information you can record in your database/paper records.

Do you record HearLab information?

☐ Yes ☐ No

If yes, please indicate how you routinely record* the following information about HearLab, by selecting one of the following:

- **Routinely recorded in an electronic field**
- **Routinely recorded in notes only (either electronically or on paper)**
- **Not routinely recorded**

***'routinely record' means that your organisation usually records this information for every child, if it is available.**

	Routinely recorded in an electronic field	Routinely recorded in notes only (paper or electronic)	Not routinely recorded
**Levels (55/65/75)**			
**Detected/not detected**			
**Aided/unaided**			
**Other**			

(if other) What other HearLab information do you record?

Questions for hearing implant services

Please indicate how you routinely records* the following information, by selecting one of the following:

- **Routinely recorded in an electronic field**
- **Routinely recorded in notes only (either electronically or on paper)**
- **Not routinely recorded**

***'routinely record' means that your organisation usually records this information for every child, if it is available.**

Information about the child

	Routinely recorded in an electronic field	Routinely recorded in notes only (paper or electronic)	Not routinely recorded
**Client status (active/lost etc)**			
**Referral date**			
**Referral reason**			
**Site/centre attended**			
**Age/date of first appointment**			
**Age/date of hearing loss confirmation/diagnosis**			
**Failure to attend appointment**			
**Aetiology (primary)**			
**Aetiology (secondary)**			
**Progressive or fluctuating loss**			
**Comorbidities**			
**Communication approach (e.g. Auslan, spoken language)**			
**Parent participation or engagement**			
**COSI/goal setting**			

Amplification information

	Routinely recorded in an electronic field	Routinely recorded in notes only (paper or electronic)	Not routinely recorded
**Age/date of initial fitting (right ear/left ear)**			

**Current amplification device(s) (e.g. type, model)**			
**Previous amplification device(s)**			
**Hearing device serial number(s)**			
**Programmed hearing aid parameters**			
**Hearing device compliance/use/datalogging**			
**Real ear measurements**			
**Coupler gain**			
**Aided audiogram**			

Hearing implant information (e.g. cochlear implant, bone anchored hearing aid)

	Routinely recorded in an electronic field	Routinely recorded in notes only (paper or electronic)	Not routinely recorded
**Pre-implantation audiology results**			
**Date hearing implant evaluation**			
**Hearing implant candidacy (eligible/ineligible)**			
**Hearing implant recommendation (e.g. type of implant/unilateral/bilateral)**			
**Family decision (e.g. proceed/don't proceed)**			
**Reason for not proceeding with implant**			
**Hearing implant surgery date(s)**			
**Hearing implant switch on date(s)**			
**Implanted device (e.g. electrode array, abutment)**			
**Hearing implant processor**			
**Hearing implant use/compliance**			
**Mode (e.g. unilateral/bilateral/bimodal)**			

**Aided audiogram (with implant)**			

Other hearing device information

	Routinely recorded in an electronic field	Routinely recorded in notes only (paper or electronic)	Not routinely recorded
**Remote microphone system fitted**			
**Remote microphone system use**			
**Other accessory or assistive listening device usage/details (e.g. app)**			

At what time points does your service record the following information on children who are deaf or hard of hearing?

	Does not record at any time	1 year post implant	2 years post implant	School entry	Other time points
**Speech perception/recognition**					
**Speech production/intelligibility**					
**Receptive language**					
**Expressive language**					
**Vocabulary**					
**Literacy**					
**Pragmatic communication**					
**Functional listening**					
**Listening effort**					
**Auditory integration**					
**Parent child interaction**					
**Cognition (IQ)/ problem solving**					
**Quality of life/Hearing related quality of life**					
**Other**					

(If other) What other pre- or post-implantation evaluations do you perform/record, and when?

Does your organisation record the details of any pre-implantation diagnostic audiology tests?

☐ Yes ☐ No

Questions for early childhood intervention and allied health services

What services do you provide? (select all that apply)

☐ Audiology

- [ ] Speech pathology
- [ ] Psychology
- [ ] Counselling
- [ ] Social work
- [ ] Physiotherapy
- [ ] Occupational therapy
- [ ] Play therapy
- [ ] Optometry
- [ ] Playgroup
- [ ] Parent support services
- [ ] Kindergarten/preschool program
- [ ] Childcare/kindergarten visits
- [ ] Home visits
- [ ] Auslan specific services
- [ ] Tele-intervention
- [ ] Other

(If other) What other service(s) do you provide?

Please indicate whether your organisation routinely records* the following information by selecting one of the following:

- **Routinely recorded in an electronic field**
- **Routinely recorded in notes only (either electronically or on paper)**
- **Not routinely recorded**

***'routinely record' means that your organisation usually records this information for every child, if it is available.**

	Routinely recorded in an electronic field	Routinely recorded in notes only (paper or electronic)	Not routinely recorded
**Location of service/facility (if multiple)**			
**Date of referral**			
**EI start date/first appointment**			
**EI discharge date**			
**Last date of engagement**			
**Patient type/caseload**			
**Which services/programs utilised**			
**Primary service provider**			
**Communication mode**			

**Current therapy plan**			
**Therapy plan outcome**			
**Therapy delivery method**			
**Therapy frequency**			
**Attendance/FTA**			
**Primary aetiology**			
**Secondary aetiology**			
**Additional diagnoses**			
**Name of school attending**			
**Other**			

(If other) Please indicate what other information you record, and how:

Outcomes matrix – Communication

At what ages does your service record the following information on children who are deaf or hard of hearing? (Please click the boxes that apply)

	Does not record at any age	0-12 months	1-3 years	4-5 years	6 years or older
**Speech perception/recognition (unaided)**					
**Speech perception/recognition (aided)**					
**Speech intelligiblity**					
**Receptive language**					
**Expressive language**					
**Vocabulary**					
**Literacy**					
**Pragmatic communication**					
**Functional listening**					
**Listening effort**					
**Auslan proficiency/fluency**					
**Parent Child Interaction**					

Outcomes matrix – Development

At what ages does your service record the following information on children who are deaf or hard of hearing? (Please click the boxes that apply)

	Does not record at any age	0-12 months	1-3 years	4-5 years	6 years or older
**Early Development Profile/ Screen**					

**Behaviour**					
**Cognition (IQ)/problem solving**					
**Executive function**					
**Memory**					
**Health Related Quality of Life**					
**Developmental/adaptive behaviour**					
**Social/emotional development**					
**Balance/vestibular function**					
**Mobility**					
**Other (please specify)**					

(If other) What other information does your service collect?

☐

Please indicate if you maintain details of any of the following in a <u>summary or report of current speech, language and communication status.</u>

- ☐ Communication mode
- ☐ Communication outcome
- ☐ Device compliance
- ☐ Receptive language diagnosis category
- ☐ Receptive language severity rating
- ☐ Expressive language diagnosis category
- ☐ Expressive language severity rating
- ☐ Speech diagnosis category
- ☐ Speech severity rating
- ☐ Functional listening outcome
- ☐ Other

(If other) Please indicate what other information is included in the summary or report of current speech, language, and communication status.

☐

Please indicate how your organisation records summary data for current speech, language and communication status.

- ☐ Routinely recorded in electronic fields
- ☐ Routinely recorded in notes only
- ☐ Not routinely recorded

Questions for medical services

Type of service: (select all that apply)

- ☐ ENT
- ☐ Paediatrics
- ☐ Genetics
- ☐ Other

(If other) Please specify ☐

This service is: (select all that apply)

- ☐ Public
- ☐ Private

Medical record type:

- ☐ Electronic
- ☐ Both electronic & paper
- ☐ Paper only

Data collected about the child

Please indicate whether your organisation routinely records* the following information by selecting one of the following:

- **Routinely recorded in an electronic field**
- **Routinely recorded in notes only (either electronically or on paper)**
- **Not routinely recorded**

***'routinely record' means that your organisation usually records this information for every child, if it is available.**

Birth history

	Routinely recorded in an electronic field	Routinely recorded in notes only (paper or electronic)	Not routinely recorded
**Delivery method**			
**APGARs**			
**Resuscitation required**			
**Admission to NICU/SCN**			
**Length of admission to NICU/SCN**			
**Need for oxygen/ventilation**			
**Birth length**			
**Birth head circumference**			
**Other birth history details**			

(If other) What other birth history details do you routinely record, and how?

Pregnancy history

	Routinely recorded in an electronic field	Routinely recorded in notes only (paper or electronic)	Not routinely recorded
**Use of fertility drugs/IVF**			
**History of recurrent miscarriages**			
**Fever during pregnancy**			

**Medications during pregnancy**			
**Antenatal investigations**			
**Other pregnancy history details**			

(If other) What other pregnancy history details do you routinely record, and how?

Medical investigations for hearing loss

	Routinely recorded in an electronic field	Routinely recorded in notes only (paper or electronic)	Not routinely recorded
**MRI brain**			
**Connexin**			
**Microarray**			
**Exome sequencing**			
**CMV (saliva/urine) at birth**			
**CMV on newborn bloodspot**			
**Renal ultrasound**			
**Thyroid function**			
**ECG**			
**Other medical investigation details**			

(If other) What other medical investigation details do you routinely record, and how?

Medical risk factors for hearing loss

	Routinely recorded in an electronic field	Routinely recorded in notes only (paper or electronic)	Not routinely recorded
**Head injury**			
**Infections requiring IV antibiotics**			
**Seizures**			
**Jaundice requiring exchange transfusion**			
**Other medical risk factors for hearing loss**			

(If other) What other details do you routinely record about other medical risk factors for hearing loss, and how?

Family history

	Routinely recorded in an electronic field	Routinely recorded in notes only (paper or electronic)	Not routinely recorded
**Childhood hearing loss**			
**Hearing loss as a young/middle-aged adult**			
**Syndrome associated with hearing loss**			
**Language delay/disorder**			
**Speech/articulation difficulties**			
**Intellectual disability**			
**Sudden death**			
**Kidney problems**			
**Cleft palate**			
**Other family history details**			

(If other) What other family history details do you routinely record, and how?

Service use

	Routinely recorded in an electronic field	Routinely recorded in notes only (paper or electronic)	Not routinely recorded
**Frequency of attendances/non-attendances**			
**Referrals**			
**Other details abotu use of your service**			

(If other) What other details about use of your service do you routinely record, and how?

Other health service use

	Routinely recorded in an electronic field	Routinely recorded in notes only (paper or electronic)	Not routinely recorded
**ENT**			

**Paediatrician**			
**Ophthalmology**			
**Early intervention**			
**Hearing implant service**			
**Other service(s)**			

(If other) What details about use of other services do you routinely record, and how?

Diagnoses and assessments

	Routinely recorded in an electronic field	Routinely recorded in notes only (paper or electronic)	Not routinely recorded
**Medical diagnosis for hearing loss (primary)**			
**Date of diagnosis**			
**Medical diagnosis for hearing loss (secondary)**			
**Medical diagnoses (other)**			
**Developmental diagnoses**			
**Developmental screening**			
**Developmental assessments**			
**Language assessments**			
**Cognitive assessments**			
**Autism assessments**			
**Other screening or assessments**			

(If other) What other screens or assessments do you use?

Audiology Diagnostic Summary (for right ear and left ear)
Please indicate if you routinely record* the following information, by selecting one of the following:

- **Routinely recorded in an electronic field**
- **Routinely recorded in notes only (either electronically or on paper)**
- **Not routinely recorded**

***'routinely record' means that your organisation usually records this information for every child, if it is available.**

	Routinely recorded in an electronic field	Routinely recorded in notes only (paper or electronic)	Not routinely recorded
**Permanent, temporary, unknown**			
**Type of hearing loss (SNHL, ANSD, permanent conductive, mixed)**			
**Atresia/Microtia (yes/no)**			
**Degree of hearing loss (e.g. mild, moderate, severe, profound)**			
**3 or 4 Frequency Average Hearing Loss**			

Hearing rehabilitation summary

	Routinely recorded in an electronic field	Routinely recorded in notes only (paper or electronic)	Not routinely recorded
**Audiology centre attended**			
**Left ear date of fitting**			
**Right ear date of fitting**			
**Hearing device compliance/use**			
**Progressive or fluctuating hearing loss**			
**Remote microphone device fitted**			
**Remote microphone device use**			
**Date of hearing implant evaluation**			
**Outcome of hearing implant evaluation**			
**Hearing implantation date(s)**			

Questions for Education Providers (Schools, Preschools, Education Departments, Regional education offices, Teachers of the Deaf)

Are you completing this survey on behalf of:

- ☐ A state or regional level education department, service or organisation
- ☐ An individual school/preschool service
- ☐ Other

(If other) Please specify ____________________

What ages/year levels do you offer services to? (Select all that apply)

- ☐ Preschool

☐ Primary
☐ Secondary

What is the setting of your program? (Select all that apply)

☐ Visits into a mainstream setting
☐ Special program for deaf or hard of hearing children within a mainstream setting
☐ Setting for deaf and hard of hearing children only
☐ Setting for children with various additional needs (e.g. special school)
☐ Tele-intervention
☐ Other

(If other) Please specify ______

What programs do you offer? ______

Data collected about the child
Please indicate whether your organisation routinely records* the following information, by selecting one of the following:

- **Routinely recorded in an electronic field**
- **Routinely recorded in notes only (either electronically or on paper)**
- **Not routinely recorded**

***'routinely record' means that your organisation usually records this information for every child, if it is available.**

	Routinely recorded in an electronic field	Routinely recorded in notes only (paper or electronic)	Not routinely recorded
**Location of service/facility (if multiple)**			
**Settings/programs utilised**			
**Support Plan (e.g. Individual Education Plan or Behaviour Support Plan)**			
**Attendance/absence data**			
**Hearing, speech, vision, mobility, or other diagnoses (e.g. obtained from school enrolment questionnaire/CASES21)**			
**Speech rating**			
**Expressive language rating**			
**Receptive language rating**			

**Pragmatic language rating**			
**Functional listening rating**			
**Listening fatigue**			
**Quality of life**			
**Behaviour problems**			
**Literacy**			
**Numeracy**			
**Cognition (IQ)**			
**Developmental/adaptive behaviour**			
**Social/emotional development**			
**Mental health**			
**NAPLAN participation**			
**Year 12 completion**			
**Student exit date**			
**Student exit reason**			
**Post-school destination**			

Do you record additional diagnoses (including intellectual disability, learning difficulty, vision impairment, Language Disorder, Specific Learning Disorder (SLD- Reading, SLD- Written Expression, SLD- Mathematics), Social Emotional Disorder (Autism Spectrum Disorder, Depression, Anxiety, Eating Disorder, Post Traumatic Stress Disorder, Obsessive Compulsive), Attention Disorder (Attention Deficit Hyperactivity Disorder, Attention Deficit Disorder), Behavioural Disorder/Conduct Disorder, Drug or Alcohol Problems)?

☐ Yes ☐ No

(If yes) How and where do you record these diagnoses?

If a school, does your school contribute data to the Nationally Consistent Collection of Data on School Students with Disability (NCCD)? https://www.nccd.edu.au/

☐ Yes ☐ No ☐ Not applicable

(If yes) What database does your school enter the NCCD data into?

(If yes) To your knowledge, has this data been used or linked to other data sets for research or reporting?

☐

Questions for Maternal and Child Health Services

Do you do hearing checks?

☐ Yes ☐ No

(If yes) At what age(s) do you check hearing?

☐

(If yes) What hearing screen do you use?

☐

Do you do vision checks?

☐ Yes ☐ No

(If yes) At what age(s) do you check vision?

☐

(If yes) What vision screen do you use?

☐

Do you do developmental screens?

☐ Yes ☐ No

(If yes) At what age(s) do you do developmental screens?

☐

(If yes) What developmental screen(s) do you use?

☐

Questions for Aboriginal and Torres Strait Islander health or hearing services

Do you provide any hearing health services specifically for Aboriginal and/or Torres Strait Islander families that you have not already told us about in the previous sections above?

☐

Please indicate whether your organisation routinely records* the following information by selecting one of the following:

- **Routinely recorded in an electronic field**
- **Routinely recorded in notes only (either electronically or on paper)**
- **Not routinely recorded**

***'routinely record' means that your organisation usually records this information for every child, if it is available.**

	Routinely recorded in an electronic field	Routinely recorded in notes only (paper or electronic)	Not routinely recorded
**Referral date**			
**Referral reason**			
**Facility name or location (if multiple)**			
**Last date of engagement**			
**Attendance/FTA**			
**Discharge from service date**			
**Patient type/caseload**			
**Primary service provider**			
**Which services/programs utilised**			

Do you do child hearing checks?

☐ Yes ☐ No

(If yes) At what age(s) do you check hearing?

(If yes) What hearing screen do you use?

Do you do child communication screens?

☐ Yes ☐ No

(If yes) At what age(s) do you do communication screens?

(If yes) What communication screen(s) do you use?

Do you do child vision checks?

☐ Yes ☐ No

(If yes) At what age(s) do you check vision?

(If yes) What vision screen do you use?

Do you do developmental screens?

☐ Yes ☐ No

(If yes) At what age(s) do you do developmental screens?

☐

(If yes) What developmental screen(s) do you use?

☐

Questions for peer-to-peer support services and health professional parent support (post newborn hearing screening)

What parent or family support services or programs does your organisation offer?

☐ Programs/services for parents
☐ Programs/services for children
☐ Programs/services for families
☐ Online forums
☐ Other

Are your programs offered by (select all that apply):

☐ Qualified health professionals
☐ Paid parent mentors
☐ Volunteer parent mentors
☐ Facilitating staff members
☐ Other

(If other) You selected Other. Please specify. ☐

Data collected about the child

Please indicate if you routinely record* the following information by selecting one of the following:

- **Routinely recorded in an electronic field**
- **Routinely recorded in notes only (either electronically or on paper)**
- **Not routinely recorded**

***'routinely record' means that your organisation usually records this information for every child, if it is available.**

	Routinely recorded in an electronic field	Routinely recorded in notes only (paper or electronic)	Not routinely recorded
**Interpreter needed**			
**Referral reason**			
**Referral date**			
**Psychosocial risk factors**			
**Case status**			

**Date of first contact (SMS, letter or phone call)**			
**Date of parent first engagement with support service**			
**Home visit**			
**Attend diagnostic audiology appointment with parent**			
**Attend Hearing Australia visit with parent**			
**Attend medical visit with parent**			
**Send resources**			
**Total time spent (supporting family)**			
**Final contact date**			
**Level of engagement (never, rarely, sometimes, often, always)**			
**Last date of engagement**			
**Reason for exiting program**			
**Audiology/hearing status**			
**Name of EI agency**			
**Other**			

(If other) Please indicate what other information you record, and how:

Questions for Deaf advocacy and support services

What services does your organisation offer for children? (select all that apply where the service is available to children aged 18 years or under)

- ☐ NDIS applications
- ☐ NDIS plan management
- ☐ NDIS support co-ordination
- ☐ NDIS appeals
- ☐ NDIS support work (please specify)
- ☐ Individual advocacy
- ☐ Interpreter services
- ☐ Auslan classes/courses
- ☐ Allied health assessment or therapy (please specify)

☐ Programs/services for Deaf or hard of hearing children (please specify)
☐ Programs/services for parents and families of Deaf or hard of hearing children (please specify)
☐ Other (please specify)

(If any of the 'please specify' options have been ticked) Please provide more information about the services your organisation provides.

Data collected about the child
Please indicate if you routinely record* the following information by selecting one of the following:

- **Routinely recorded in an electronic field**
- **Routinely recorded in notes only (either electronically or on paper)**
- **Not routinely recorded**

***'routinely record' means that your organisation usually records this information for every child, if it is available.**

	Routinely recorded in an electronic field	Routinely recorded in notes only (paper or electronic)	Not routinely recorded
**Date of first engagement**			
**Case status**			
**Communication approach (e.g. Auslan, spoken language)**			
**Interpreter needed**			
**Services received/program enrolments**			
**Dates services received**			
**Level of engagement (never, rarely, sometimes, often, always)**			
**Audiology/hearing status**			
**Name of EI agency**			
**Other (please specify)**			

(If other) Please indicate what other information you record, and how:

Questions for research registries or longitudinal cohorts

Please describe your research registry or longitudinal cohort.

What other information does your organisation/service record that might be relevant to a national childhood hearing databank?

☐

ANCHOR Survey Part One C

Data Governance

Does your organisation obtain specific written consent from a parent/guardian when they use/enrol in your service/program?

☐ Yes ☐ No

If yes, please upload a copy of your consent/terms of service form, or email it to anchor@mcri.edu.au.

How does your organisation/service manage changes to consent, withdrawal of consent or constraints around who can provide consent?

☐

Does your organisation include a privacy or data use statement in your terms of service or research (for example, a statement on your website)?

☐ Yes ☐ No

If yes, please copy the statement into the box or include a website link

☐

Does your organisation participate in research activities? (Please select one option)

☐ We are a research organisation or university
☐ We deliver services and have an internal research arm
☐ We deliver services and have an affiliation with a research institute or university (please specify)
☐ We deliver services and participate in research activities when approached by external organisations
☐ We do not participate in research activities
☐ Other

If yes to affiliation, please specify

☐

How does your organisation manage data and research requests? (Please select all that apply)

☐ We have an internal ethics committee
☐ We have an internal research committee or approval process
☐ Data/research requests are managed by an external organisation (please specify)
☐ We do not have a process for data/research requests

If yes to managed by an external organisation, please specify

☐

Please describe any other data governance issues we need to be aware of (e.g. specific considerations for Aboriginal and Torres Strait Islander data sovereignty)

☐

Is there a specific piece of legislation that governs your organisation for handling and use of data?
If yes, please provide the name of the piece of legislation.
If no, please indicate which other pieces of legislation your organisation follows for the collection and use for personal and outcome data e.g., *Health Records Act 2001 (Vic), Privacy and Data Protection 2014 (Vic), Privacy Act 1988 (Cth)*

☐ Yes ☐ No ☐ Not sure

If yes, please provide the name of the piece of legislation

If no, please indicate which other pieces of legislation your organisation follows

Where is your database located?

☐ On premises ☐ Cloud ☐ Don't know

If cloud, where is it hosted?

☐ Australia ☐ Overseas ☐ Don't know

If overseas, which country is it hosted in?

Who maintains control over security and privacy? (Select one)

☐ Database admininstrator
☐ Company IT department
☐ Specialist data security office
☐ Other – please specify

If other, please specify

Data Linkage

How would you rate the quality of your data in terms of completeness, presences of known errors, degree of curation?

☐ Poor
☐ Moderate
☐ Acceptable
☐ Good
☐ Excellent
☐ Don’t know

☐ Yes

Has your organisation participated in any previous data linkage project e.g. for a clinical trial or other population health research?

☐ No
☐ Don't know

If yes, please provide a description of data linkage projects your organisation has been involved in.

What appetite is there within your organisation to understand and undertake data linkage activities?

Thank you for completing Part One.

ANCHOR Survey: Part Two

Part Two

About costs, including the estimated cost of staffing, data collection, data entry and database management and the potential cost of data systems change.

This part comprises of a spreadsheet that we will send via email. Part two can be completed at a later date by a staff member who has information about staff salaries and other costs.

Please nominate the name and contact details of someone within your organisation who we can contact about Part Two. This can be you, or someone else.

**Name**	
**Role**	
**Email address**	
**Phone number**	

Please let them know that we will be in touch soon. You may download the invitation letter for Part Two to send the nominated person if you wish.

Attachment: Part two intro email Costing analysis for ANCHOR.docx

Part Three

For services in Victoria and Queensland only

About the database/s and data governance. This section asks technical questions about your organisation's databases. This section may be completed most easily by a data/database manager or IT officer, however if your organisation does not have a specific data person, please fill out the questions as best you can.

This section has 18 questions and will take about 15 minutes to complete.

Would you like to continue to Part Three or nominate another staff member from your organisation to complete Part Three?

☐ I would like to continue to Part Three
☐ I would like to nominate another staff member to complete Part Three

(If selected would like to nominate another staff member) Please nominate the name and email address of someone within your organisation who we can contact about Part Three:

**Name**	
**Role**	
**Email address**	
**Phone number**	

Please press Submit to finish, your colleague will be contact about Part Three. Please let them know to expect contact from the ANCHOR team. Thank you!

(If selected continue to Part Three) Please press Submit to continue to Part Three.

ANCHOR Survey: Part Three

Part Three

About the database/s and data governance. This section asks technical questions about your organisation's databases. This section may be completed by a data/database manager or IT officer, however if your organisation does not have a specific data person, please fill out the questions as best you can. We hope to use information from Part Three to plan data linkage between Victorian and Queensland data sets.

All your responses will be kept confidential and no organisation will be identified in any publication of results. You and your organisation will have the opportunity to view the report of results prior to publication.

If you have any questions or prefer to complete the questionnaire in an online meeting format with researchers, please contact us by email anchor@mcri.edu.au.

This section will take about 15 minutes to complete.

(If completed by a different staff member, they will be asked to complete the consent page also. If continued by same staff member, the consent question will be skipped)

By ticking this box and continuing with the survey, I consent to:

1. **Participation in the Australian National Child Hearing health Outcomes Registry (ANCHOR) Aim 1 Survey.**

2. **Use of the information I provide:**
    a. **To help determine the feasibility of a National Child Hearing health Outcomes Registry**
    b. **To help determine a core set of outcomes to be measured for Australian deaf and hard of hearing children**
    c. **To plan data linkage between databases (Victorian and Queensland), OR to inform the development of a model for a future national registry (other states).**
3. **To be contacted by the ANCHOR team if they need to clarify any answers or need more information**

☐ Yes

Database overview

In Part 1 of this survey you/colleague provided information about the following database/s: [platform name]

What platform does [platform name] run on?

☐ MySQL
☐ PostgreSQL
☐ SQL Server
☐ Oracle
☐ Access
☐ Excel
☐ Other
☐ Don't know

(If other) please specify the platform

Do you have a data dictionary for this database?

☐ Yes ☐ No ☐ Don't know

(If yes) Can you provide us with a copy of your data dictionary?

☐ Yes ☐ No

If yes, what format is it in, and is it readily accessible to data users?

(If no/don't know) Do you have a list/schema of data variables collected?

☐ Yes ☐ No ☐ Not sure

How regularly is your database software updated?

☐ Every six months
☐ Every 12 months
☐ Every 2 years
☐ 2-5 years
☐ Over five years
☐ Don't know

Are there plans to migrate the database to a new platform in the future?

- ☐ No
- ☐ Within six months
- ☐ 6-12 months
- ☐ 12-24 months
- ☐ Over three years
- ☐ Don't know

(If answered any of the options except no) As part of a database migration, would all data be brough across or only a subset e.g. data within the last five years

- ☐ All data
- ☐ Subset of data
- ☐ None – start new database from scratch
- ☐ Don't know

(If answered subset of data) How many years of data will be included?

Data quality

Do you undertake any formal data quality assessments? If so, what does this entail and how often are they completed?

☐ Yes ☐ No ☐ Don't know

If so, what does this entail and how often are they completed?

What processes are in place for addressing identified data quality issues e.g., missing or incorrect names and demographic information, invalid or erroneous data?

What tools are used for cleaning data?

- ☐ Data cleaning not routinely performed
- ☐ Python
- ☐ Strata
- ☐ R
- ☐ Other
- ☐ Don't know

(If other) Please indicate what other tools are used for cleaning data.

Who is responsible for cleaning data? (Please select all that apply)

- ☐ Data scientist
- ☐ Statistician
- ☐ Database administrator

☐ Other

(If other) Please indicate what other tools are used for cleaning data.

☐

Data Variables

What volume of data on children with hearing loss is held in your database (e.g., total number of records held on children 0-18 years, number of new records per year)?

☐

What metadata is maintained for the database? Metadata is information about other data without including the actual value for the data item of interest e.g., date and time a value was recorded, unit of measurement for the data item, whether the data item is formatted as a number or text.

☐

Is there a metadata catalogue available?

☐ Yes ☐ No ☐ Don't know

Is the database built using a common data model (CDM) such as Observational Medical Outcomes Partnership (OMOP)? If no, what is the data model based on?

☐ Yes ☐ No ☐ Don't know

If yes, please describe the model.

☐

If no, what is the data model based on?

☐

Is a standard ontology (data naming convention and relationships) used? e.g., SNOMED, or has the database been developed from historical paper forms?

☐ Standard ontology ☐ Developed from paper forms ☐ Don't know

Has a privacy impact assessment (PIA) been carried out for your database?

☐ Yes ☐ No ☐ Don't know

If yes, when?

☐

Does your organisation have a database manager or other technical support staff? Are these services contracted out, or employed by your organisation? (Select all that apply)

☐ Dedicated database manager – directly employed
☐ Shared by general technical support staff – directly employed
☐ Contracted database support service
☐ Contracted IT provider

☐ Other

(If other) Please specify

Does your organisation have a preferred model for providing data to a researcher or for data linkage?

☐ Yes ☐ No ☐ Don't know

If yes, what does this involve?

